# Translational approaches to influence sleep and arousal

**DOI:** 10.1016/j.brainresbull.2022.05.002

**Published:** 2022-05-10

**Authors:** Ritchie E. Brown, Tristan J. Spratt, Gary B. Kaplan

**Affiliations:** a Laboratory of Neuroscience, VA Boston Healthcare System and Harvard Medical School, Dept. of Psychiatry, 1400 VFW Parkway, West Roxbury, MA 02132, USA; b Mental Health Service, VA Boston Healthcare System, West Roxbury, MA, 02312, USA; c Departments of Psychiatry and Pharmacology & Experimental Therapeutics, Boston University School of Medicine, Boston, MA 02118, USA

**Keywords:** CBT-I, Deep brain stimulation, Non-invasive stimulation, Pitolisant, Suvorexant, Zolpidem

## Abstract

Sleep disorders are widespread in society and are prevalent in military personnel and in Veterans. Disturbances of sleep and arousal mechanisms are common in neuropsychiatric disorders such as schizophrenia, post-traumatic stress disorder, anxiety and affective disorders, traumatic brain injury, dementia, and substance use disorders. Sleep disturbances exacerbate suicidal ideation, a major concern for Veterans and in the general population. These disturbances impair quality of life, affect interpersonal relationships, reduce work productivity, exacerbate clinical features of other disorders, and impair recovery. Thus, approaches to improve sleep and modulate arousal are needed. Basic science research on the brain circuitry controlling sleep and arousal led to the recent approval of new drugs targeting the orexin/hypocretin and histamine systems, complementing existing drugs which affect GABA_A_ receptors and monoaminergic systems. Non-invasive brain stimulation techniques to modulate sleep and arousal are safe and show potential but require further development to be widely applicable. Invasive viral vector and deep brain stimulation approaches are also in their infancy but may be used to modulate sleep and arousal in severe neurological and psychiatric conditions. Behavioral, pharmacological, non-invasive brain stimulation and cell-specific invasive approaches covered here suggest the potential to selectively influence arousal, sleep initiation, sleep maintenance or sleep-stage specific phenomena such as sleep spindles or slow wave activity. These manipulations can positively impact the treatment of a wide range of neurological and psychiatric disorders by promoting the restorative effects of sleep on memory consolidation, clearance of toxic metabolites, metabolism, and immune function and by decreasing hyperarousal.

## 1. Introduction

A large body of important work links disrupted sleep and arousal mechanisms to a variety of mental and physical disorders. Thus, here we review the current state-of-the-art of research into translational approaches to influence sleep and arousal. We begin by summarizing the basic features of sleep-wake control that are needed to understand different translational approaches. We emphasize the importance of basic sleep and circadian research and illustrate recent bench-to-bedside success stories. We subdivide interventions into behavioral approaches, pharmacological treatments, non-invasive stimulation approaches and invasive approaches. Subsequently, we discuss approaches which may improve sleep induction, sleep duration, depth of sleep and enhance specific sleep features to promote the beneficial aspects of sleep such as memory consolidation and clearance of toxic metabolites. We do not cover the effects of exercise, yoga, meditation, or diet, although these manipulations may also have beneficial effects on sleep quality. We also do not cover environmental/societal changes such as alterations in school start times, ambient lighting, or noise pollution although these are also important approaches grounded in empirical science. Treatments for specific sleep disorders involving disordered breathing or abnormal muscle or movement control are also not covered. The focus is on approaches which can promote healthy sleep-wake cycles and boost or correct specific cortical electrical oscillations occurring in different sleep-wake states. We emphasize that the approaches described here are not direct recommendations for clinical practice but rather represent a summary of current, future, and blue-sky research approaches. We hope that this comprehensive review will be helpful for basic and translational researchers, engineers, chemists, pharmacologists, and clinicians trying to improve the lives of those with disorders of sleep and arousal. Box 1 provides our views of important questions for future translational work.

## 2. The importance of sleep and arousal for mental and physical health

Sleep can be considered one of the three pillars of healthy living, together with a good diet and exercise. Conversely, disrupted sleep is one of the most common complaints that patients report to their primary care physicians. Population level data suggest that approximately 6% of the adult US population has been diagnosed with a sleep disorder, representing 14 million adults (Huyett and Bhattacharyya, 2021). Using this conservative prevalence rate, the incremental health care cost of sleep disorders in the US was estimated to be almost $100 billion (Huyett and Bhattacharyya, 2021). Moreover, sleep disturbances-related productivity loss and absenteeism in the workplace lead to financial costs for corporations. A 2010 survey of 4188 employees across 4 US companies estimated this cost to be $1967 per employee each year (Rosekind et al., 2010).

Sleep and arousal disturbances are extremely common in those with traumatic brain injuries (Griesbach and Rowe, 2022; Landsness et al., 2011; Thibaut et al., 2019), neurodegenerative diseases (Eugster et al., 2016; Katsuki et al., 2022; McCleery and Sharply, 2020) or psychiatric disorders (Baglioni et al., 2016; Benca et al., 1992; Krystal, 2019). Some neuropsychiatric disorders exhibit deficits in particular sleep features which may interfere with restorative effects of sleep. For instance, a selective deficit in sleep spindle density and the coupling of sleep spindles to slow oscillations has been observed in schizophrenia (Ferrarelli, 2021; Manoach and Stickgold, 2019), a feature which may disrupt sleep-dependent memory consolidation. Improving sleep quality leads to better mental health, even in those without a diagnosis of a mental disorder (Scott et al., 2021). Extreme disturbances of arousal networks occur in patients with disorders of consciousness (Snider et al., 2019; Thibaut et al., 2019), even though some aspects of circadian and sleep-wake function may be preserved (Landsness et al., 2011). Thus, a wide range of brain disorders involve a sleep or arousal component.

The authors of this article are based at the Veterans Administration (VA) Boston Healthcare System. Sleep and arousal disorders are prevalent in active military personnel and in Veterans. Deployment and combat exposure substantially increase the risk for insomnia and other sleep disorders in military personnel (Caldwell et al., 2019; Colvonen et al., 2020; Folmer et al., 2020; Good et al., 2020). These findings correlate with the large increase in the diagnosis of sleep disorders in the VA in recent years (Alexander et al., 2016; Martin et al., 2020). Disturbances of sleep and arousal are common in disorders which are areas of emphasis for the VA such as traumatic brain injury (TBI), post-traumatic sleep disorder (PTSD), depression, suicide and substance abuse (Baglioni et al., 2016; Benca et al., 1992; Bishop et al., 2020; Chakravorty et al., 2018; Griesbach and Rowe, 2022; Leng et al., 2021; McCarthy et al., 2019; Ressler et al., 2022; Stavitsky Gilbert et al., 2015). Hyperarousal and disturbing dreams which disrupt sleep are major features of PTSD and antagonizing brain arousal systems may be beneficial (Kaplan et al., 2022; Ressler et al., 2022; Stavitsky Gilbert et al., 2015). In a large study of almost 200,000 US Veterans, those with TBI were 41% more likely to be diagnosed with a sleep disorder, independent of a PTSD diagnosis (Leng et al., 2021). Sleep disturbance is associated with an increased risk for suicide in US veterans (McCarthy et al., 2019) and in non-veterans (Geoffroy et al., 2021; Hedström et al., 2021) independent of the presence of an underlying psychiatric disorder (Geoffroy et al., 2021). Insomnia is also commonly observed during withdrawal from alcohol (Sharma et al., 2010, 2022) and from opioids (Chakravorty et al., 2018) and contributes to increased risk for relapse. Furthermore, successful treatment of sleep disturbances improves chronic pain in veterans (Saconi et al., 2021). Thus, improving sleep and decreasing hyperarousal are likely to have many beneficial effects for the Veteran population (Folmer et al., 2020), for active service members, as well as for the civilian population (Parthasarathy et al., 2016).

## 3. The value of basic science for translational research

The NIH National Center for Advancing Translational Sciences defines translation as “the process of turning observations in the laboratory, clinic, and community into interventions that improve the health of individuals and populations – from diagnostics and therapeutics to medical procedures and behavioral interventions”. Funding agencies are understandably focused on translational approaches to justify the public investment in biomedical science. However, the bedrock for translational approaches is basic, curiosity driven science which uncovers the biology underlying normal and pathological states. The sleep-wake field has some of the best examples of the value of this approach. In the 1980 s, a classic pharmacological approach led to the identification of a new receptor for histamine, the H_3_ receptor, an auto and heteroreceptor which inhibited the release of histamine and other arousal-promoting neurotransmitters (Schwartz, 2011). Thus, antagonism of this receptor would be expected to promote arousal by increasing the brain levels of histamine and other wakefulness-promoting neurotransmitters (Brown et al., 2001b). Consistent with this prediction, an antagonist/inverse agonist of this receptor, pitolisant, was recently approved for the treatment of narcolepsy with cataplexy and for hypersomnia (Abramowicz et al., 2021; Schwartz, 2011; Szakacs et al., 2017).

Another fine example of basic science leading to novel treatments of sleep and arousal is the discovery of the orexin/hypocretin system, its’ link to narcolepsy and the development of drugs targeting this system (Brown, 2003; Herring et al., 2019; Kuriyama and Tabata, 2017; Wurts Black et al., 2017). The search for ligands binding orphan G-protein linked receptors and for novel peptides expressed in the hypothalamus led to the identification of new peptide neurotransmitters, named the orexins or hypocretins by two separate groups (De Lecea et al., 1998; Sakurai et al., 1998) and to the discovery of the two receptors to which they bind and exert their effects. Very rapidly a link of the orexin/hypocretin system to the sleep disorder narcolepsy was established through experiments where the preproorexin gene was knocked out in mice (Chemelli et al., 1999) and a gene defect was discovered in the orexin/hypocretin type II receptors in a colony of dogs with genetically determined narcolepsy (Lin et al., 1999). Subsequent experiments revealed a loss of orexin/hypocretin neurons in postmortem brains of narcolepsy patients and a reduction of CSF orexin/hypocretin levels in living narcolepsy patients (Nishino et al., 2000; Peyron et al., 2000; Thannickal et al., 2000). Thus, destruction of orexin/hypocretin neurons was discovered as the main cause of human narcolepsy. Subsequent pharmaceutical drug development led to dual orexin receptor antagonists (DORAs) which promote sleep (Gotter et al., 2016) and are approved for the treatment of insomnia (Herring et al., 2018). Ongoing pharmaceutical work shows promise in developing orexin receptor agonists to promote wakefulness and counteract excessive daytime sleepiness (Ishikawa et al., 2020; Yukitake et al., 2019). Thus, basic science identifying this neurotransmitter system rapidly led to the identification of the pathology underlying narcolepsy, as well as drug development.

Although the pathway from basic science discovery to development of approved treatments is slow, encompassing 20–30 years, these two strong examples show the value of this approach in providing treatments with novel mechanisms of action which are grounded in rational science. We firmly believe that further research into the genes, proteins, neurons, and brain circuitry controlling sleep and wakefulness is essential in developing additional treatments. Next, we briefly summarize current knowledge of sleep-wake circuitry as a springboard to understanding current and future treatment approaches.

## 4. Brief summary of sleep-wake control circuitry and mechanisms which can be targeted by translational approaches

Subjective assessments and questionnaire-based approaches are commonly used in clinical practice and research to assess sleep. Heart rate variability-based sleep trackers and actigraphy are also being used more and more in basic and clinical research. Nonetheless, polysomnography (EEG/EMG) still represents the gold standard for sleep assessment in human and mammalian sleep research and provides a powerful tool to decipher changes in sleep-wake physiology (Prerau et al., 2017). Wakefulness is characterized by increased low amplitude, fast (>4 Hz) activity in the EEG and high skeletal muscle tone in the EMG. Low amplitude, fast activity in the EEG is controlled by a self-reinforcing network of ascending arousal systems from the brainstem, midbrain and hypothalamus which excites midline and intralaminar thalamic neurons projecting to superficial and deep layers of the cortex, as well as several different types of cortically projecting basal forebrain neurons (Anaclet et al., 2015; Brown et al., 2012; Gent et al., 2018a, b; Jones, 2005). Direct projections to the neocortex of these ascending arousal systems, which include cholinergic and aminergic systems (noradrenaline, serotonin, dopamine, histamine), also promote the fast cortical activity typical of wakefulness (Jones, 2005). How these systems modulate different functional cortical networks, such as the default mode network, is still poorly understood (Aguilar and McNally, 2022). Surprisingly, cell-type specific, loss-of-function studies in animals have revealed that loss of cholinergic and aminergic systems has surprisingly minor effect on sleep-wake cycles (Saper and Fuller, 2017) with the exception that cholinergic basic forebrain lesions markedly reduce homeostatic sleep rebound following sleep deprivation (Brown et al., 2012). Recent basic science work in animals has focused on the role of GABAergic and glutamatergic systems. Fast discharging cortical GABAergic interneurons and ascending subcortical GABAergic neurons which target them are key regulators of cortical gamma (30–80 Hz) activity associated with cognition and active brain states (Brown and McKenna, 2015; Gutierrez Herrera et al., 2016; Kim et al., 2015; McNally et al., 2021). Glutamatergic neurons in the basal forebrain (Xu et al., 2015), ventral tegmental area (Yu et al., 2019) and supramammillary nucleus (Pedersen et al., 2017) strongly promote wakefulness. The peptidergic orexin/hypocretin neurons described in the previous section do not affect the overall amount of sleep or wakefulness over a 24 hr period but are key regulators of consolidated wakefulness and sleep through their strong excitatory actions on many other wakefulness promoting systems (Brown et al., 2012; Saper et al., 2001).

At the beginning of the transition from wakefulness to sleep, the EEG begins to slow, showing more posterior alpha (8–14 Hz) and frontal theta (4–8 Hz) activity (Prerau et al., 2017). Two processes drive the slowing of the cortical EEG and the shutting-off of wakefulness-promoting systems, the homeostatic and circadian sleep drives (Achermann and Borbely, 2003). The homeostatic sleep drive increases in proportion to time spent awake. Homeostatic sleep factors such as adenosine and nitric oxide accumulate during prolonged wakefulness in the basal forebrain (Kalinchuk et al., 2006, 2010; Porkka-Heiskanen et al., 1997, 2000), and promote sleep by inhibition of wakefulness-promoting cholinergic and non-cholinergic basal forebrain neurons (Arrigoni et al., 2006; Yang et al., 2013). In addition to global changes in neuronal activity, increased neuronal activity during prolonged wakefulness is also associated with local cortical, use-dependent increases in sleep-like activity (Krueger et al., 2008; Vyazovskiy et al., 2011b). The circadian sleep drive is mediated by the master circadian clock located within GABAergic neurons in the suprachiasmatic nucleus of the ventral anterior hypothalamus (Reppert and Weaver, 2001). Retinal inputs to this nucleus and other non-light cues (zeitgebers) synchronize the activity of this nucleus with daily light-dark cycles. A transcriptional feedback loop within the SCN leads to ~24 hr cycles of activity (Reppert and Weaver, 2001) which are transmitted to sleep-wake control neurons via intermediate neurons in the supraventricular zone and dorsomedial hypothalamus (Saper et al., 2005). Silencing of wakefulness promoting neurons by homeostatic and circadian mechanisms leads to the disinhibition of GABAergic sleep-promoting neurons in several parts of the preoptic hypothalamus (Lu et al., 2000; Sakai, 2020; Saper et al., 2001; Sherin et al., 1996; Szymusiak et al., 1998). Additional GABAergic neurons which promote NREM sleep are located in the cortex (Gerashchenko et al., 2008; Morairty et al., 2013), lateral hypothalamus/zona incerta (K. Liu et al., 2017; M. Liu et al., 2017) and parafacial region of the brainstem (Anaclet et al., 2014).

Although most basic research has focused on the role of different neurons in regulating sleep-wake activity, in recent years increasing evidence supports that idea that not only neurons but also glia in the basal forebrain, preoptic and posterior hypothalamus and in the neocortex regulate sleep-wake cycles and sleep homeostasis (Halassa et al., 2009; Ingiosi et al., 2020; Kim et al., 2020; Pelluru et al., 2016). Thus, these cells are potential targets for sleep/arousal-modulating agents.

As sleep deepens and thalamic reticular (TRN) and thalamocortical (TC) neurons become more hyperpolarized due to the withdrawal of excitatory inputs, distinct EEG features become apparent (Brown et al., 2012; Steriade and McCarley, 2005). Waxing and waning patterns of burst discharge of TRN and TC neurons lead to sleep spindles (11–16 Hz) recorded in the cortex (Steriade and McCarley, 2005), a characteristic feature of stage 2 sleep in humans. Synchronized burst discharge of TRN neurons, especially the subset which contain the calcium-binding protein, parvalbumin (Clemente-Perez et al., 2017; Thankachan, Katsuki et al., 2019), leads to sleep spindles by generating a rapid hyperpolarization in TC neurons which leads to rebound bursting, which is transmitted to the cortex and back to the TRN, leading to a new oscillatory cycle (Steriade and McCarley, 2005). An increase in sleep spindles occurs prior to the transition to REM sleep. Optogenetic studies in mice suggest that this increase promotes NREM→REM transitions (Bandarabadi et al., 2020).

As sleep deepens further, slow-wave activity (0.5–4 Hz) becomes dominant in the EEG, a combination of largely cortically generated slow waves (0.5–1.5 Hz) and thalamically generated delta (1.5–4 Hz) oscillations. Slow waves (0.5–1.5 Hz) originate in the frontal cortex and move posteriorly as a travelling wave (Massimini et al., 2004), coordinating the timing of other NREM sleep oscillations (Crunelli and Hughes, 2010; Steriade et al., 1993). Recent evidence suggests that the claustrum, a poorly understood area of the forebrain with abundant connections to cortex and thalamus, may be especially important in coordinating cortical slow wave and hippocampal sharp wave-ripple activity during NREM sleep (Narikiyo et al., 2020; Norimoto et al., 2020) but to date this area has not been a focus of translational studies. Delta oscillations (1.5–4 Hz) are largely thalamically generated due to the interplay of intrinsic voltage-gated channels in TC neurons which become active with increased hyperpolarization (Jahnsen, Llinás, 1984a, 1984b; Dossi et al., 1992; Uygun and Basheer, 2022). Slow-wave activity (0.5–4 Hz) during sleep, especially in the delta2 (2.5–3.5 Hz) band, is a marker of sleep intensity and increases following periods of sleep deprivation or disruption (Hubbard et al., 2020). Slow wave activity decreases during the night in humans and during extended sleep periods in animals, a reflection of synaptic homeostasis mechanisms (Vyazovskiy et al., 2011a). The precisely coordinated nesting of sleep spindles, slow oscillations and also hippocampal ripples (70–200 Hz) is implicated in the memory consolidation functions of sleep (Klinzing et al., 2019). Slow waves and delta oscillations are implicated in other important restorative functions of sleep in regulating cellular metabolism (Wisor et al., 2013), DNA repair (Bellesi et al., 2016; Everson et al., 2014), immune function (Besedovsky et al., 2019) and the clearance of proteins implicated in neurodegenerative disorders (Fultz et al., 2019; Xie et al., 2013).

A distinct phase of sleep linked to vivid dreams was discovered by Aserinsky, Dement and Kleitman in humans and named rapid-eye-movement (REM) sleep (Aserinsky & Kleitman, 1953; Dement and Kleitman, 1957). The equivalent sleep-wake state was discovered by Jouvet and colleagues in animals (Jouvet, 1962; Jouvet et al., 1965) and named ‘paradoxical sleep’ since the EEG was more similar to wakefulness than to NREM sleep, even though the skeletal muscle tone is absent, apart from during phasic twitches. Transection and lesion studies established the upper brainstem (dorsolateral pons) as the key area responsible for the generation of REM sleep (Jouvet et al., 1965). Reciprocal interaction models involving REM active and REM-inactive neurons nicely described the time course of alternation of NREM and REM sleep during the night in humans (McCarley and Hobson, 1975). Cholinergic and glutamatergic brainstem neurons were proposed as the REM promoting/REM active neurons in these models, whereas noradrenergic and serotonergic neurons were REM-inhibiting/REM-off (McCarley, 2007). More recent models downplay the importance of cholinergic neurons and ascribe important roles to GABAergic neurons (Lu et al., 2006b; Luppi et al., 2013). Although REM is generated in the upper brainstem it is nonetheless regulated by neurons in other brain areas, such as the lateral hypothalamus orexin/hypocrein and melanin-concentrating hormone (MCH) neurons.

Beyond rapid-eye-movements themselves, several signature features of the REM state are of importance. High amplitude electrical events called PGO waves can be recorded from the pons, lateral geniculate nucleus of the thalamus and occipital cortex, suggesting a role in visual dreams (Datta, 1997; Steriade and McCarley, 2005). Correlates of these events can also be observed in the hippocampus and other brain areas and may be involved in synaptic plasticity/memory consolidation processes occurring during REM sleep (Datta et al., 2008). Muscle atonia is a key feature of REM sleep which normally prevents us from acting out our dreams but gets activated during wakefulness in cataplexy attacks in narcolepsy (McKenna and Peever, 2017). REM atonia is generated by a small group of glutamatergic neurons located just ventral to the locus coeruleus in cats (SubC) and higher mammals and just ventral to the lateral dorsal tegmental cholinergic neurons in rodents (SubLDT or SLD). These large neurons project to inhibitory glycinergic and GABAergic neurons in the ventral medulla and spinal cord which in turn inhibit motoneurons. They are susceptible to degeneration, leading to REM sleep behavior disorder (RBD), a precursor of alpha-synucleiopathies such as Parkinson’s disease (Gagnon et al., 2006; McKenna and Peever, 2017). High-amplitude continuous theta (4–8 Hz) activity is typical of REM sleep in rodents and linked to memory consolidation (Buzsaki, 1989) but theta activity in humans tends to be more intermittent and of a slightly slower frequency (Cantero et al., 2003).

This short summary of the physiology sets the stage to allow us to discuss how best to modulate the brain mechanisms and circuitry which controls sleep and arousal. For a more in-depth discussion of the neurons, genes and proteins which control sleep and wakefulness please see other more comprehensive reviews (Brown et al., 2012; Luppi and Fort, 2019; Saper and Fuller, 2017; Schwartz and Kilduff, 2015; Steriade and McCarley, 2005).

## 5. Behavioral approaches

Cognitive behavioral therapy (CBT-I) is the first-line treatment recommended for insomnia by the American Academy for Sleep Medicine. CBT-I is effective in promoting sleep initiation and maintenance through a combination of approaches which promote sleep hygiene, address counterproductive thought processes and enhance sleep pressure i.e. the homeostatic sleep drive (Boland et al., 2019; Parthasarathy et al., 2016). CBT-I is a safe, effective and durable treatment for many patients with sleep disruption (Parthasarathy et al., 2016). Although CBT-I is normally conducted with a trained therapist, emerging evidence supports the use of CBT-I conducted via telemedicine as effective (Boland et al., 2019; Folmer et al., 2020). Ongoing studies are investigating how to best combine CBT-I with other behavioral or non-behavioral approaches to improve outcomes in major depression, substance abuse, obesity, TBI, PTSD and other disorders (Boland et al., 2019; Folmer et al., 2020; Pigeon et al., 2019; Stavitsky Gilbert et al., 2015; Trockel et al., 2015). Although safe and effective, CBT-I does not help all patients, normally requires a therapist trained in CBT-I and is unlikely to be helpful for disorders which involve disruption of specific aspects of sleep-wake physiology. Furthermore, the sleep restriction component of CBT-I is not recommended for patients with seizure disorder or bipolar depression (Boland et al., 2019). Thus, other complementary approaches are needed.

## 6. Pharmacological approaches

Although CBT-I is the recommended first-line treatment for insomnia, pharmacological approaches are commonly used to treat sleep disorders, especially in the short-term. Thus, in this section we review currently available options, subdivided by their target, as well as promising future candidates.

### 6.1. Benzodiazepine and non-benzodiazepine receptor allosteric agonists of synaptic GABA_A_ receptors

The most widely prescribed hypnotic agents are those which potentiate the activity of the major inhibitory neurotransmitter in the brain, gamma-amino-butyric acid (GABA), acting on GABA_A_ receptors. GABA_A_ receptors exist as hetero-pentameric, ligand-gated ion channels and conduct chloride ions following activation by GABA, which results in hyperpolarization, inhibition of neuronal discharge and synchronization of neuronal ensembles in the thalamocortical system. Sleep-promoting neurons in the preoptic hypothalamus, lateral hypothalamus and brainstem use GABA as their primary neurotransmitter (Anaclet et al., 2014; Brown et al., 2012; Liu et al., 2017; Lu et al., 2000, 2006b; Saper et al., 2001). GABAergic neurons in several parts of the basal ganglia also strongly regulate the amount of sleep (Lazarus et al., 2013; Qiu et al., 2010). Thus, pharmacological agents which potentiate the activity of GABA_A_ receptors, including benzodiazepines receptor agonists (lorazepam, clonazepam, diazepam, etc.) and the non-benzodiazepine receptor agonists known as the Z-drugs (Zolpidem, zopliclone etc.), facilitate sleep initiation and slightly increase sleep duration (Krystal et al., 2013). However, these drugs also affect GABA_A_ receptors throughout the brain, including those which promote fast oscillations during wakefulness and REM sleep and the thalamocortical oscillations typical of NREM sleep (Brown and McKenna, 2015). Thus, as discussed by Uygun and Basheer (2022), they disrupt healthy sleep architecture, reducing deep NREM sleep and enhancing light NREM sleep. Interestingly, these drugs increase the density of sleep spindles and can promote sleep-dependent memory consolidation (Zhang et al., 2020), which may be advantageous in conditions such as schizophrenia which exhibit sleep spindle abnormalities (Mylonas et al., 2020), as long as the coupling of spindles to the upstate of cortical slow oscillations is maintained. However, their suppression of deep sleep stages which promote clearance of toxic proteins from the brain and synaptic downscaling is likely to be detrimental for overall brain health. Furthermore, these drugs can lead to dependence, falls, increased daytime sleepiness and delirium in older patients. Thus, they need to be used with care and for short periods only.

Compared to earlier hypnotics, benzodiazepines have proven to be safer in the management of acute and chronic insomnia. However, since benzodiazepines occupy GABA_A_ receptors throughout the brain, side effects, include memory impairments, psychomotor slowing, balance problems and addiction potential which can restrict their long-term use. As a non-benzodiazepine receptor agonist, zolpidem serves as one of the most popular “Z” drugs and its pharmacology and clinical effects have been well reviewed (Becker and Somiah, 2015). Zolpidem increases the affinity of GABA to GABA_A_ receptors and produces longer chloride channel openings and greater duration of GABAergic function. Compared to other hypnotics acting on GABA_A_ receptors, zolpidem has higher affinity for GABA_A_ receptors containing the alpha1 subunit, thought to be the main sleep promoting GABA_A_ receptor complex, but it also has affinity for GABA_A_ receptor complexes containing other alpha subunits (Fitzgerald et al., 2014; Kralic et al., 2002). Zolpidem binds to receptors in a variety of regions: sensorimotor cortex, cerebellum, olfactory bulb, ventrolateral preoptic complex of thalamus, inferior colliculus, globus pallidus and other regions which produce its wide-spread effects including memory loss and psychomotor slowing. It is rapidly absorbed after oral administration and has a shorter half-life than many benzodiazpines (2.2 h). As such, it achieves rapid peak plasma levels. Zolpidem reduces sleep latency, increases sleep efficiency and number of awakenings, improves next day functioning, and is approved for sleep onset and maintenance insomnia with fewer effects on next day performance if taken at night. It is optimally used for single or intermittent use. A notable problem with zolpidem is that it can induce dependency, and in rare cases be abused. As mentioned, anterograde amnesia as well as short-term memory loss are also concerns, and other undesirable effects like somnambulism, sleep eating, sleep driving and other purposeful behaviors have been reported. With chronic use, even in the elderly, the risk of mortality was not significantly associated with hypnotic use regardless of the type and duration. However, falls and accidents can be problems, especially in an elderly population (Jaussent et al., 2013). Intriguingly, dramatic paradoxical arousal effects of zolpidem have been reported in a subset of patients with disorders of consciousness which exhibit increased alpha-band (8–12 Hz) activity (Williams et al., 2013; Thibaut et al., 2019). This effect of zolpidem may be due to the high level of GABA_A_ alpha1 subunits in basal ganglia output neurons which are overactive (Schiff, 2010).

### 6.2. Extrasynaptic GABA_A_ receptor agonists

In addition to mediating phasic GABAergic synaptic transmission, GABA can also elicit a tonic suppression of neuronal activity mediated by high-affinity receptors containing α4, α6 and delta subunits. Activation of these receptors with a selective agonist promotes slow-wave activity in rodent brains (Mesbah-Oskui et al., 2014; Winsky-Sommerer et al., 2007). These receptor subunits are highly expressed in the thalamus, an important controller of sleep-wake activity and cortical oscillations (Houston et al., 2012; Mesbah-Oskui et al., 2014). Accordingly, there has been considerable interest in selective agonists of these receptors as potential hypnotics or promoters of sleep slow oscillations. Unfortunately, clinical trials of one such agent, gaboxadol, were unsuccessful (Wisden et al., 2017). Extrasynaptic GABA_A_ receptors are also one molecular target of the hypnotic agent, gamma-hydroxybutyric acid (GHB)(Absalom et al., 2012). Several anticonvulsants may promote sleep through GABAergic mechanisms, enhancing the effect of GABA at synaptic and extra-synaptic GABA_A_ receptors and GABA_B_ receptors (Krystal et al., 2013). Tiagabine blocks GABA re-uptake whereas gabapentin and pregabalin increase GABA concentrations at synapses by acting on voltage-gated calcium channels, actions which may lead to spillover of GABA onto extrasynaptic receptors. They may be used for improving sleep in the context of treatment of other comorbid neuropsychiatric disorders (Atkin et al., 2018).

### 6.3. Agonists of GABA_B_ receptors

The other main receptor for GABA is the G-protein coupled, GABA_B_ receptor. Activation of GABA_B_ receptors generates a long-lasting and deep hyperpolarization of target neurons through activation of G-protein coupled inwardly rectifying potassium (GIRK) channels and inhibits synaptic release from GABAergic and non-GABAergic neurons. GHB (Xyrem™) is an FDA-approved treatment for narcolepsy which improves sleep continuity and reduces cataplexy through mechanisms that are not well understood. Although other mechanisms may contribute to its sleep-promoting effect, activation of GABA_B_ receptors on wakefulness-promoting neurons is likely to be a major mechanism of action. In healthy male human volunteers, GHB prolonged NREM sleep at the expense of REM sleep (Dornbierer et al., 2019). In marked contrast to allosteric agonists of GABA_A_ receptors, GHB enhanced slow, delta and theta activity, while reducing activity in the sleep spindle range, showing similarities with the properties of recovery sleep following a period of sleep deprivation (Dornbierer et al., 2019), even though the subjects were not sleep deprived. GHB has abuse potential so is only recommended for narcolepsy and hypersomnia.

### 6.4. Orexin receptor antagonists

Anatomical studies identified the location of orexin/hypocretin neurons in the perifornical regions of the lateral hypothalamus and their strong projections to wakefulness-promoting and REM suppressing neurons (De Lecea et al., 1998; Marcus et al., 2001; Peyron et al., 1998; Sakurai et al., 1998), whereas electrophysiological experiments revealed the strong excitatory effects of orexins/hypocretins on aminergic, cholinergic and other wake-promoting systems (Brown et al., 2001a, 2002, 2012; Burlet et al., 2002; Eggermann et al., 2001; Eriksson et al., 2001; Korotkova et al., 2003). *In vivo* pharmacological and molecular biology experiments supported the wakefulness promoting and REM suppressing effects of this system (summarized in Brown, 2003; Brown et al., 2012). As discussed in Section 3, orexin receptor antagonists were developed as sleep-promoting agents as a direct result of basic science research. Dual orexin receptor antagonists (DORAs) show efficacy in animal and human studies of insomnia and are approved by the US FDA (Herring et al., 2019). The first agent to be approved, Suvorexant, decreased sleep onset latency and improved total sleep time and wake time after sleep onset (WASO) versus placebo (Herring et al., 2019; Kuriyama and Tabata, 2017). Sleep maintenance effects were stronger than those on sleep latency. The increase in total sleep time was found to be due to increases in all sleep stages, although REM sleep was preferentially increased at higher doses, consistent with the REM suppressing effects of the orexin/hypocretin system observed in animal models. The EEG microstructure was not affected, reflecting a more natural sleep pattern than observed with GABAergic drugs. Common side effects were daytime somnolence and abnormal dreams (Herring et al., 2019; Kuriyama and Tabata, 2017). A comparative study of hypnotics suggested that suvorexant was more effective in reducing WASO compared to a variety of GABA_A_ receptor agents, the melatonin receptor agonist ramelteon or the tricylic antidepressant and histamine H_1_ receptor antagonist, doxepin (Zheng et al., 2020). Selective orexin type II receptor antagonists are also likely to be effective in promoting sleep (Gotter et al., 2016). The role of orexin type I receptors in sleep control is less well defined but is likely to play a REM-suppressive role based on its high expression in the locus coeruleus (Chen et al., 2010). DORAs are also being investigated for disorders of arousal such as PTSD (Kaplan et al., 2022) and delirium (Xu et al., 2020) and for improving sleep in people with dementia (McCleery and Sharply, 2020). DORA have less impact on next-day cognitive and motor performance and preserve arousal to salient stimuli. In these regards, DORAs may be advantageous over traditional GABAergic medications (Coleman et al., 2017).

### 6.5. Orexin receptor agonists

Given the wakefulness-promoting and REM-suppressing actions of the activation of the orexin/hypocretin system demonstrated in basic research studies and the destruction of orexin/hypocretin neurons observed in most human cases of narcolepsy with cataplexy, it has long been hoped that orexin agonists could be developed which would alleviate the symptoms of type I narcolepsy and treat excessive daytime sleepiness and/or hypersomnolence in a variety of other disorders (Wurts Black et al., 2017). Development of these drugs proved difficult due in part to the high receptor occupancy needed to elicit functional effects. However, recently a series of orexin type II receptor agonists were developed and robust wakefulness promoting actions were demonstrated in wild-type mice (Yukitake et al., 2019) and in orexin-deficient mice (Ishikawa et al., 2020). Early clinical trials suggested promise and led to an FDA breakthrough therapy designation for an oral formulation. Unfortunately, at the time of writing, a phase 2 trial was halted due to safety concerns. One potential area of concern could be the potential for addiction since orexin/hypocretins strongly activate dopaminergic midbrain neurons (Korotkova et al., 2003). Nonetheless, this translational approach appears likely to be extremely beneficial if safety concerns can be remedied.

### 6.6. Melanin-concentrating hormone

Another important basic science advance which may have translational potential for sleep disorders was the discovery of sleep-active and sleep-promoting melanin-concentrating hormone (MCH) neurons neighboring the wake-promoting orexin/hypocretin neurons in the lateral hypothalamus (Verret et al., 2003). MCH neurons discharge fastest during sleep, especially REM sleep (Hassani et al., 2009). Chemogenetic or optogenetic stimulation of MCH neurons in rodents consistently increases REM sleep, with more variable effects on NREM sleep, dependent on the stimulation paradigm (Jego et al., 2013; Konadhode et al., 2013; Varin et al., 2018; Vetrivelan et al., 2016). Furthermore, intriguing basic science studies suggest that REM-sleep active MCH neurons are involved in forgetting hippocampus-dependent learning (Izawa et al., 2019) and modulating fear conditioning (Concetti et al., 2020). Thus, pharmacological activation of this system might be expected to both promote sleep and help in disorders such as insomnia and PTSD which involve altered REM-related processing of emotional memories (Ressler et al., 2022; Riemann et al., 2012; Van Someren, 2021).

### 6.7. Monoaminergic agents

Pharmacological agents which increase the levels of dopamine and noradrenaline are the strongest promoters of prolonged wakefulness which are currently available in the pharmaceutical armamentarium (Brown, 2016). As such, they are commonly prescribed to treat excessive daytime sleepiness in narcolepsy and other disorders. Animal studies have confirmed that activation of midbrain and brainstem dopamine neurons, as well as noradrenaline neurons promotes wakefulness (Brown et al., 2012; Jones, 2005; Saper and Fuller, 2017; Lu et al., 2006a). Furthermore, the targets of midbrain dopamine neurons in the basal ganglia strongly regulate the amount of wakefulness and sleep (Lazarus et al., 2013; Qiu et al., 2010). However, the use of psychostimulants is limited by their abuse potential and by the increase in the homeostatic sleep drive they produce (Lin et al., 2000). Modafinil also boosts dopamine release from midbrain dopamine neurons (Yang et al., 2021) but surprisingly has less pronounced effects on sleep rebound (Lin et al., 2000). Modafinil also has additional actions which enhance focused wakefulness and cognition. For instance, modafinil has effects on gap junction proteins in neurons (Garcia-Rill et al., 2007) and in astrocytes (Liu et al., 2016; Duchene et al., 2016) which may increase the arousal and pro-cognitive effects. In fact, a novel combination of modafinil with an astroglial gap junction modulator, flecainide (THN102), has shown promise in improving vigilance and cognition in healthy sleep deprived subjects and ameliorating excessive daytime sleepiness in Parkinson’s disease patients (Corvol et al., 2021; Sauvet et al., 2019). An initial study suggested that prazosin, an antagonist of alpha1 noradrenergic receptors, might be useful to treat nightmares in PTSD patients (Raskind et al., 2003). However, a more recent clinical trial showed no benefit for nightmares or for improving sleep quality (Raskind et al., 2018).

Antidepressants which act on serotonergic systems can be used to treat sleep disturbances. In particular, the antidepressant, trazodone, which antagonizes wakefulness promoting 5-HT_2A_/_2 C_ receptors is commonly prescribed off-label for the treatment of insomnia in both depressed and non-depressed patients (Leger et al., 2018). Low-dose mirtazapine, an antidepressant with 5HT2A/5HT2C/5HT3 and α2 adrenoreceptor antagonism, increased total sleep time and reduced the number of awakenings versus placebo in a trial of individuals with sleep problems (Karsten et al., 2017). Both these agents are commonly used in psychiatric practice for insomnia complaints.

### 6.8. Histaminergic drugs

The brain histamine system was relatively understudied for many years in comparison with other aminergic systems. However, the wakefulness promoting actions of this system are well established (Brown et al., 2001b). The soporific effects of histamine H_1_ antagonists which cross the blood-brain barrier are well-known and they are common ingredients in over-the-counter night-time formulations for colds and pain. Many psychiatric drugs, such as the tricyclic antidepressants, doxepin, trazodone and mirtazapine also have significant activity at histamine receptors, as well as other monoaminergic receptors and are used to treat sleep disorders (Krystal et al., 2013). As discussed in Section 3, Pitolisant is a novel, FDA approved, agent which enhances arousal and cognition through inverse agonism at H_3_ auto and heteroreceptors and ameliorates cataplexy in narcolepsy patients (Dauvilliers et al. (2013); Szakacs et In contrast to other stimulants, pitolisant has low abuse potential (Setnik et al., 2020) since the histamine system does not target the midbrain dopamine neurons which are one of the main targets of addictive drugs (Korotkova et al., 2002).

### 6.9. Adenosine receptor antagonists

The most widely used psychoactive compounds are the adenosine receptor antagonists, caffeine and theophylline, the main active ingredients in coffee and tea (Fredholm et al., 1999). Caffeine increases arousal and produces concentration-dependent improvements in cognition and motor performance in healthy adults at low to intermediate plasma concentrations and anxiety at higher concentrations (Fredholm et al., 1999; Kaplan et al., 1997). Caffeine and theophylline antagonize the adenosine A_1_ receptors in the basal forebrain and cortex that play a major role in sleep homeostasis (Basheer et al., 2004; Elmenhorst et al., 2007; Thakkar et al., 2003; Yang et al., 2013), and the adenosine A_2a_ receptors that are heavily expressed on the indirect basal ganglia pathways in the dorsal and ventral striatum (Huang et al., 2005). Both actions likely contribute to the wakefulness promoting actions of these agents (Brown et al., 2012). Interestingly, polymorphisms in adenosine-related genes markedly affect the response to caffeine and the response to sleep deprivation (Bachmann et al., 2012; Bodenmann et al., 2012).

### 6.10. Melatonin and melatonin receptor agonists

Over-the-counter melatonin is widely used to entrain circadian rhythms and to promote sleep (Cajochen et al., 2003). Melatonin and the melatonin receptor agonist, ramelteon, act on two receptors, MT_1_ and MT_2_, which play distinct roles in sleep regulation. MT_1_ receptors are highly expressed in the suprachiasmatic nucleus and mediate the effects of melatonin and melatonin agonists on circadian rhythms (Stein et al., 2020). Recently, the determination of the crystal structure of melatonin receptors and computational techniques led to the discovery of MT_1_ receptor selective ligands which are active in vivo at low doses (Stein et al., 2020). MT_1_-selective inverse agonists advanced the phase of circadian rhythms in mice by 1.3–1.5 hr when applied at subjective dusk. Thus, these agents may have potential for treating circadian rhythm disorders. MT_2_ receptors are located elsewhere in the brain, notably in the thalamic reticular nucleus (TRN). Application of a selective MT_2_ receptor agonist (UCM765) to the TRN depolarized TRN neurons and promoted deep NREM sleep (Ochoa-Sanchez et al., 2011). However, this agent has low water solubility and only modest metabolic stability (Ferlenghi et al., 2021). Thus, further development of this class of agents is needed before clinical applications.

### 6.11. Potential novel drug targets

Basic science findings suggest that there are several other potential pharmacological targets which may prove fruitful in drug discovery. Genetic studies in invertebrate systems have implicated shaker-type potassium channels that are involved in action potential repolarization as major regulators of sleep amount (Cirelli et al., 2005; Koh et al., 2008) and sleep homeostasis (Pimentel et al., 2016). Knockout of a shaker-type potassium channel alpha subunit with high expression in the thalamocortical system (Kcna2) in mice also reduced the amount of NREM sleep (Douglas et al., 2007). Leak potassium channels are major regulators of the resting membrane potential and are targets of multiple arousal-promoting neurotransmitters (Brown et al., 2012). A computational model of sleep-wake control predicted an important role for leak potassium channels in regulating sleep duration (Yoshida et al., 2018). To test this model these authors examined sleep in 14 knockout mice models. Knockout of the Kcn9 leak potassium channel decreased sleep duration. A mouse study investigating sleep instability in aging suggested that modulators of KCNQ2/3 channels may help improve sleep in older individuals by correcting the decrease in KCNQ2/3 activity in orexin/hypocretin neurons observed during aging (Li et al., 2022). The Transient receptor potential family of cation channels, known as the TRP superfamily, are also important mediators of the depolarizing effect of wakefulness promoting neurotransmitters (Sergeeva et al., 2003) and are therapeutic targets in other disorders (Koivisto et al., 2022). Collectively, these findings suggest that in addition to currently available anticonvulsants which act on various ion channels, selective manipulators of several different-types of voltage-gated ion channels could be useful in modulating sleep and arousal.

A considerable body of work suggests the importance of immune signaling in sleep-wake control, not just in disease states (Imeri and Opp, 2009) but also in the regulation of normal sleep (Krueger et al., 2001). Multiple signaling molecules involved in immune regulation affect sleep including cytokines and ATP (Krueger et al., 2001; Brown et al., 2012). As well as the being the universal energy currency of the body, extracellular ATP is an important signaling molecule. Degradation of ATP to adenosine and activation of inhibitory A_1_ adenosine receptors in the basal forebrain is an important component of the sleep homeostatic response. However, ATP also activates a large family of ionotropic (P2X) and metabotropic purinergic receptors (P2Y) located on neurons, glia, blood vessels and immune cells. Activation of P2 receptors in the basal forebrain promotes wakefulness via excitatory effects on cholinergic and cortically projecting GABAergic neurons (Yang et al., 2018), whereas activation of P2X_7_ receptors promotes sleep (Krueger et al., 2010), possibly by actions on glia in the cortex, which as discussed in Section 4 are emerging as important regulators of sleep.

Trace amine receptors are an interesting novel pharmacological target to treat disorders of sleep and wakefulness (Black et al., 2017). Recently, a partial agonist (R05263397) of trace-amine associated receptor increased wakefulness in macaques without any impairment of cognition (Goonawardena et al., 2019).

### 6.12. Summary of pharmacological approaches to treat sleep and arousal

Sleep promotion by administration of benzodiazepine and non-benzodiazepine allosteric agonists of GABA_A_ receptors is well-established in clinical practice, as are the use of low-dose antidepressants such as trazodone and mirtazapine (Atkin et al., 2018). An agent which acts on GABA_B_ receptors, GHB, is approved to promote sleep and treat cataplexy in narcolepsy patients. Basic science studies suggest that agonists of extrasynaptic GABA_A_ receptors are also likely to be effective in promoting deep sleep if safe, brain-penetrating agents can be identified. Dual orexin receptor antagonists are a recent addition to the clinical arsenal which were developed based on basic science discoveries. These agents appear to promote a more natural sleep than GABA_A_ receptor agonists, with less side effects. Melatonin or melatonin receptor agonists are approved to correct circadian rhythms and promote sleep, especially in the elderly. Basic science studies suggest that melanin concentrating hormone receptor agonists could be useful sleep-promoting agents.

Caffeine and theophylline are widely used to promote wakefulness, but their use is limited by tolerance and withdrawal effects (Fredholm et al., 1999). Polymorphisms in adenosine metabolism and receptors affect the response to these agents. Psychostimulants are strong wake-promoting agents but can be abused and lead to a strong sleep rebound. Modafinil promotes focused wakefulness with less sleep rebound. The histamine H_3_ receptor inverse agonist, pitolisant, is a novel wakefulness and pro-cognitive agent which has recently been approved in the US and has less abuse potential. Orexin receptor agonists are likely to be extremely beneficial to treat narcolepsy and hypersomnia if safe and effective agents can be developed.

## 7. Non-invasive stimulation approaches

**Fig. 1.**
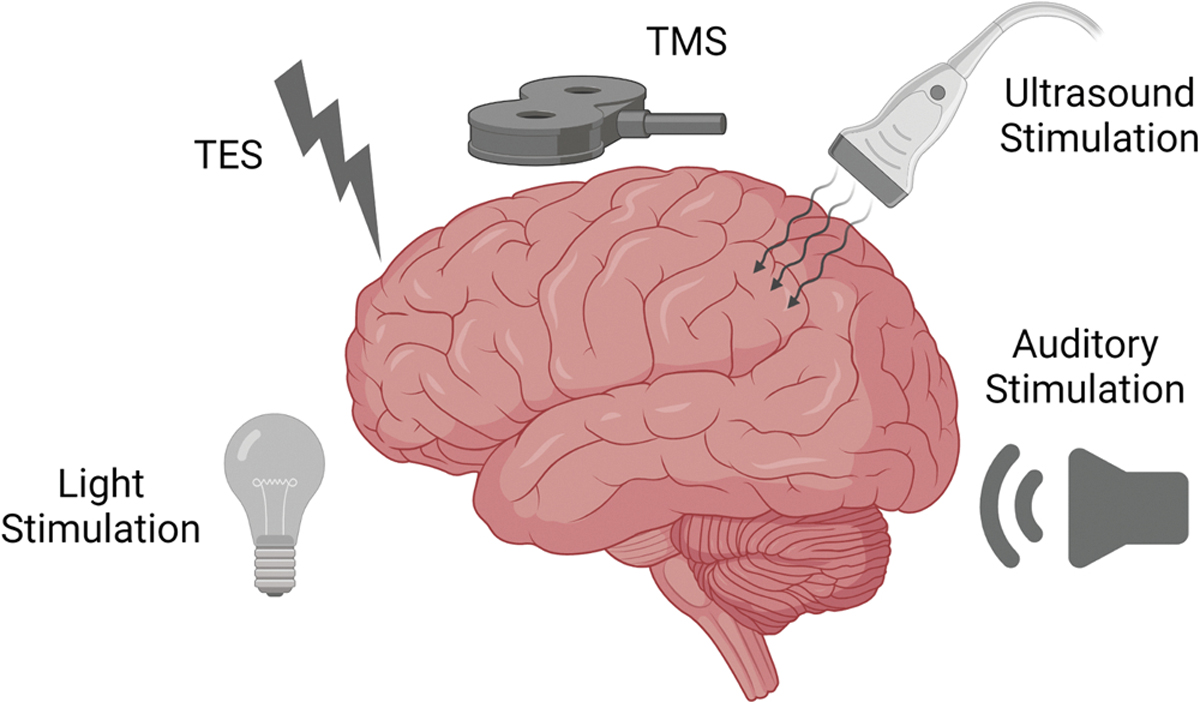
Non-invasive stimulation approaches to influence sleep and arousal. Sleep, arousal, and the cortical oscillations characteristic of different sleep-wake states can be manipulated through a variety of techniques. Transcranial electrical stimulation (TES) and transmagnetic stimulation (TMS) techniques increase, decrease, or entrain the activity of specific cortical areas and indirectly affect subcortical circuits. Low-intensity ultrasound can be targeted to deep brain areas such as the thalamus or basal forebrain. Activation of sensory systems using light, sound, or vestibular stimulation (not shown) alters arousal and cortical oscillations via relays in the hypothalamus and brainstem which in turn alter the activity of ascending arousal pathways to the cortex. Figure created with BioRender.com.

There is great interest in non-invasive brain stimulation approaches due to their safety and applicability to both healthy and patient populations. A variety of approaches have been proposed (Fig. 1), which may help promote sleep timing with respect to the light-dark cycle, extend sleep duration/depth or promote specific features of sleep. Unlike chemical agents that influence bodily function by flooding the entire bloodstream, non-invasive brain stimulation techniques can directly target strategic brain regions through the skin and cranium or manipulate sensory pathways to influence neuronal activity while minimizing undesired side effects. In this section we briefly explain how these stimulating techniques work and describe prominent studies which have used these techniques to target arousal or sleep.

### 7.1. Transcranial electrical stimulation (TES)

Transcranial electrical stimulation refers to non-invasive brain stimulation techniques that generate a weak electrical current and deliver it into the brain to alter neuronal excitability. In practice, TES is usually delivered through two or more electrodes strategically positioned on the scalp to target specific brain regions (Reed and Cohen Kadosh, 2018). TES can be further subcategorized into different groups based on the type of electrical current that is used. Transcranial direct current stimulation (tDCS) delivers a polarized direct current into the brain. A tDCS montage is always composed of at least one positive electrode called the anode, and one negative electrode called the cathode. The electrical current generated by tDCS can be anodal (flowing from the anode to the cathode), or cathodal (flowing from the cathode to the anode), which determines the polarity of the stimulation. Anodal stimulation increases neural firing rates by depolarizing the targeted neurons, while cathodal stimulation decreases firing rates by hyperpolarizing them (Liebetanz, 2002). tDCS therefore has a tonic influence: it can increase or decrease spontaneous neuronal firing by depolarizing or hyperpolarizing the resting membrane potential (Nitsche et al., 2008). Transcranial alternating current stimulation (tACS) is similar to tDCS in most aspects since it delivers a weak electrical current to stimulate specific areas of the brain. However, tACS uses a current that oscillates back and forth between two opposite amperage values, usually in a sine wave pattern. This type of stimulation has a rhythmic influence as the naturally occurring cortical oscillations in the targeted region entrain to the stimulating frequency (Tavakoli and Yun, 2017) which can increase the power of that specific frequency in the brain. Using tACS could therefore allow researchers to modulate the wavelengths and amplitudes of cortical oscillations in a safe and effective way, but the task is more complicated in practice due to the instability of cortical oscillations over time (Lafon et al., 2017). To benefit from both the tonic influence of tDCS and the rhythmic influence of tACS, direct and an alternating current can be combined to form a polarized alternating current. The resulting stimulation method is usually called oscillating transcranial direct current stimulation (osc-tDCS). Finally, a fourth type of stimulation called transcranial random noise stimulation (tRNS) which generates a current that randomly alternates between frequencies within a certain range can also be used to alter neural excitability, but we found no studies that examined the effects of this type of TES in sleep research.

Some evidence supports the idea that TES can promote sleep onset and/or the EEG oscillations typical of the transition to sleep in healthy subjects (D’Atri et al., 2016). Anodal osc-tDCS induces sleepiness in healthy subjects when applied with a theta frequency and is surprisingly more effective at enhancing endogenous delta activity in this frequency range than in the delta frequency range (D’Atri et al., 2016). Similarly, applying tACS to the fronto-temporal brain regions with a current alternating in the theta range increases the spectral power of theta and alpha brain waves when compared to sham (D’Atri et al., 2017). Although D’Atri and colleagues (2017) found no effect on self-reported sleepiness, these results suggest that using non-invasive methods to boost brainwaves associated with sleepiness may help accelerate sleep onset.

A month-long tDCS treatment also decreased sleep onset latency (SOL) in a small study of people with insomnia (Jung and Jun, 2019). TES has also been used during sleep to try to increase total sleep time (TST), as described next. Frase et al. (2016) delivered tDCS to healthy subjects by placing two anodes on the frontal lobe and two cathodes on the parietal lobe. Participants received anodal, cathodal, and sham stimulation over the course of 3 nights separated by 1 week each, and a polysomnographic recording was taken on the nights following each stimulation to measure their TST. However, anodal stimulation *decreased* TST compared with cathodal and sham stimulation settings and cathodal stimulation did not increase TST. Based on their findings from the 2016 study on healthy individuals described above, Frase et al. (2019) tested the same tDCS montage and protocol on 19 individuals with insomnia disorder. No change in TST was found in any of the stimulation conditions in this experiment. Thus, tDCS alone does not appear to be effective in increasing TST. In contrast, slow oscillatory tDCS does increase SWA (Marshall et al., 2004) and improves sleep quality (Saebipour et al., 2015). Saebipour and colleagues delivered 0.75 Hz osc-tDCS and sham stimulation on 6 individuals with insomnia in a within-subjects design over two nights. Two anodes were placed on the frontal lobe and two cathodes on the mastoids. Stimulation or sham was administered once the subjects entered their first stage 2 of sleep. Analyzing the polysomnographic recording of participants’ sleep following the stimulation period revealed the following improvements: a 9% increase in sleep efficiency, which is defined by the ratio of total sleep time over total time in bed, associated with a 12% decrease in transitions from stage 2 of sleep to wakefulness (Saebipour et al., 2015). On top of improvements on TST described above, Saebipour et al. (2015) also analyzed NREM slow-wave activity (SWA)-enhancements of their 0.75 Hz osc-tDCS montage. They found that, compared to sham, osc-tDCS caused a robust increase in the duration of deep, stage 3 NREM sleep. On average, osc-tDCS increased the duration of stage 3 of NREM by 33 min, decreased stage 1 of NREM by 22 min, and decreased sleep fragmentation such that the duration of stage 1 sleep and wakefulness after sleep onset together decreased by 55.4 min (Saebipour et al., 2015). These results are very encouraging in the possibility of using slow osc-tDCS to help people with insomnia return to baseline levels of SWA during sleep. In summary, alternating current tES methods like tACS and osc-tDCS appear most promising in promoting sleep onset. Osc-tDCS also shows promise in promoting sleep oscillations and their coupling in mild cognitive impairment (Ladenbauer et al., 2017) and in other disorders (Malkani and Zee, 2020) but to date there are relatively few studies with small sample sizes. TES can be used to elicit sleep spindles and has been effectively used to modulate memory consolidation during sleep (see Section 9.7.). TES is generally considered very safe with only minor side effects such as headaches, itching or burning feelings around the electrodes (Malkani and Zee, 2020).

### 7.2. Transcranial magnetic stimulation (TMS)

TMS devices generate magnetic pulses by passing short bursts of electrical current through an electromagnetic coil. In contrast to TES, TMS elicits more powerful and long-lasting effects on neuronal activity and can lead to synaptic plasticity (Malkani and Zee, 2020). TMS pulses usually last approximately 100 microseconds and can reach up to 2 Tesla (Hallett, 2007). When TMS is delivered directly above the head, these magnetic pulses can penetrate the skin and skull to reach the cerebral cortex where a secondary electrical current is induced parallel to the one in the coil (Kobayashi and Pascual-Leone, 2003). The magnetic field created by TMS can therefore be thought of as a bridge that allows the electrical current that passes through the coil to generate a parallel stimulating electrical current in the targeted region of the cerebral cortex. The investigators who use TMS to influence neural activity during sleep often use a technique called repetitive TMS (rTMS) in which magnetic pulses with a set intensity are generated repeatedly over time, at a frequency of at least 1 per second. Lower stimulation frequencies (around 1 Hz) tend to reduce cortical excitability while higher frequencies (around 20 Hz) lead to temporary increases in cortical excitability (Kobayashi and Pascual-Leone, 2003). TMS has been thoroughly investigated as a potential treatment method for various neuropsychiatric disorders. As of today, the FDA has approved TMS technologies as viable treatments for major depressive disorder, obsessive-compulsive disorder and pain associated with migraine headaches (Office of the Commissioner, 2018). Several studies have used TMS in sleep research, although at present it appears to be primarily a research tool rather than a potential clinical tool due to the need for a technician and a variety of technical issues associated with the device needed to generate the TMS pulses (Malkani and Zee, 2020). Nonetheless, an increasing number of studies are investigating its effectiveness for sleep disturbances in a variety of neurological and psychiatric conditions. For systematic review of TMS and TES sleep studies see (Feher et al., 2021; Herrero Babiloni et al., 2021).

In a pioneering study, Massimini et al. (2007) used TMS to increase slow cortical oscillations and sleep spindles in the cortex of healthy subjects. The stimulating current was delivered at 4 different midline locations along the anterior-posterior axis during phase 2 of NREM sleep with a frequency of approximately 0.8 Hz. Their TMS montage successfully increased the amplitude of spindles, and triggered SWA which started directly under the location of the TMS coil and diffused in all directions along the cortex (Massimini et al., 2007). Of note, only 6 out of the initial 15 participants’ data was analyzed since only these 6 subjects stayed asleep for at least one full block of stimulation (40 * 4 pulses). However, these results still suggest that TMS has the potential to trigger spindles and increase the duration of slow-wave sleep stages by inducing SWA in the cortex.

Protocols that expose participants with insomnia disorder to more rTMS treatments over longer periods may be more effective in combatting sleep disturbances. For example, Jiang et al. (2013) evenly split 120 participants with insomnia into three groups. They selected participants who experienced high levels of daytime sleepiness, and significant difficulty falling and staying asleep, following the widely used definition of Primary Insomnia disorder from the American Psychiatric Association’s Diagnostic and Statistical Manual (DSM-IV). The first group received rTMS treatment every day over a period of 2 weeks, while the second and third received medication treatment and psychotherapy respectively over the same period. The medication treatment group took 2 mg of estazolam every night, and the psychotherapy treatment group received cognitive behavioral therapy. These authors used a 1 Hz rTMS on the right dorsolateral prefrontal cortex (DLPFC) of subjects in the first group. Among other positive results, the investigators found a significant increase in TST in all three condition groups after analyzing the data from a nighttime polysomnographic test after the 2-week treatment period. Moreover, sleep efficiency significantly increased in all three groups. These results suggest that rTMS was at least as effective at increasing total sleep time as medication and psychotherapy (Jiang et al., 2013)! Total sleep time and sleep efficiency were also increased for people with both major depression and insomnia following repeated tDCS to the dorsolateral prefrontal cortex compared to sham (Zhou et al., 2020). Interestingly, this increase in sleep quality was paired with improvements in self-reported depression and anxiety measures (using the self-rating depression scale and the self-rating anxiety scale). Of note, all 90 participants in this study were undergoing a drug treatment combining Escitalopram and Zopiclone which are prescribed for depression and insomnia respectively (Zhou et al., 2020).

### 7.3. Low-intensity focused ultrasound

Although the cortex plays an important role in the generation of brain electrical activity and may be manipulated in a top-down manner to control sleep (Krone et al., 2017), many of the neurons which most powerfully control sleep, wakefulness and cortical oscillations lie deep in the brain (Section 4). The non-invasive approaches discussed above only indirectly affect these regions through manipulations of cortical electrical activity under the skull. Ideally one would like to directly modulate the activity of these deep brain areas. One technique which may enable this is low-intensity ultrasound stimulation (Meng et al., 2021). Ultrasound has been used for many years for imaging and high-intensity ultrasound can be used to lesion tumors or particular brain areas (Meng et al., 2021). In contrast, low-intensity ultrasound waves can be focused to target deep brain areas and modulate neuronal activity, likely by modulating mechanically sensitive or thermally sensitive ion channels. This technique has successfully been used to target the cortex, as well as areas involved in sleep-wake control such as the ventral tegmental area in mice (Bian et al., 2021; Qiu et al., 2020), basal forebrain in monkeys (Khalighinejad et al., 2020) and the thalamus in pigs (Dallapiazza et al., 2018), sheep (Kim et al., 2021) and in humans. Thus, it has the potential to modulate arousal or sleep. To the best of our knowledge, low-intensity ultrasound has not been used to modulate sleep so far but in rodent studies low-intensity ultrasound stimulation of the thalamus (Yoo et al., 2011) or ventral tegmental area (Bian et al., 2021) facilitated recovery from anesthesia and human studies targeting the thalamus showed promise in a small number of patients with disorders of consciousness (Monti et al., 2016; Cain et al., 2021). Interestingly, low-intensity ultrasound can also be used to temporarily increase blood-brain barrier permeability to deliver viral vectors or other therapeutic compounds to select brain regions (Meng et al., 2021; Wang et al., 2017). Thus, low-intensity focused ultrasound is an intriguing technology that has the potential to influence sleep, awareness and arousal in several different ways.

### 7.4. Auditory stimulation to promote sleep or sleep oscillations

Many people use white noise or recordings of natural sounds such as rain to facilitate falling asleep, although the evidence for its effectiveness appears weak (Riedy et al., 2021). Auditory stimulation is a non-invasive method that can influence sleep since investigators are able to use rhythmic acoustic stimuli or stimuli timed to the phase of ongoing sleep oscillations to influence sleep architecture (Bellesi et al., 2014; Malkani and Zee, 2000)(Wunderlin et al., 2021). In auditory stimulation, subjects listen to sound stimuli which have been specifically tailored to cause a change in neural activity while and/or before they fall asleep. Auditory stimuli are very effective in inducing K complexes and slow oscillations during NREM and the majority of auditory stimulation studies have been targeted to increase slow wave activity or spindle-slow wave complexes (Malkani and Zee, 2020). However, auditory stimulation can also be tailored to induce sleep spindles at specific frequencies (Antony and Paller, 2017) or to induce PGO waves during REM sleep (Bellesi et al., 2014; Malkani and Zee, 2020; Wunderlin et al., 2021).

In a landmark study, Ngo et al. (2012) examined the possibility of using auditory stimulation to induce slow-wave activity. Healthy subjects were split into three condition groups such that participants either listened to tones at a frequency of 0.8 Hz, tones separated by random time intervals, or no tones at all. The acoustic stimulation started in wakefulness and carried on for 90 min into sleep. Results showed that the subjects in the 0.8 Hz auditory stimulation group’s sleep was altered in two ways compared to the other two groups. First, it took them significantly longer to fall asleep, and second, these participants showed increased SWA spectral power once they managed to fall asleep and only while the tones were being played (Ngo et al., 2012). Auditory stimulation could therefore be used as an effective tool to enhance SWA if it can be strategically triggered after sleep onset so that listeners don’t have trouble falling asleep. Auditory stimulation also enhanced slow-wave activity during daytime naps in healthy subjects (Simor et al., 2018), and during nighttime sleep for people with mild cognitive impairment (Papalambros et al., 2019).

Auditory stimulation, and other non-invasive stimulation approaches, often use sleep, sleep oscillations or sleep-dependent memory consolidation as outcome measures (Section 9.7.). However, some studies suggest that auditory stimulation of sleep oscillations can have other beneficial effects. Besedovsky et al. (2017) found that auditory closed-loop stimulation of EEG enhanced sleep slow oscillations in men and reduced T and B cell counts, likely by redistributing these cells to lymphoid tissue. Grimaldi et al. (2019) similarly found that auditory stimulation increased parasympathetic activity during sleep, as assessed by heart rate variability and reduced overnight changes in cortisol levels. Thus, auditory stimulation induced enhancement of slow wave activity can strengthen sleep-autonomic system interactions and the immune supportive function of sleep. Thus, this type of non-invasive stimulation could have potential benefits in cardiovascular and other disorders.

### 7.5. Vestibular stimulation

All parents know that rocking and bouncing are good way to help a baby to fall asleep (Schreiner and Staresina, 2019). Furthermore, many people, especially children, fall asleep easily in cars and other modes of transportation. Recently, scientists have re-examined the mechanisms by which rocking impacts sleep and sleep physiology. In a mouse study (Kompotis et al., 2019), lateral rocking during the light (sleep) period increased time spent in NREM sleep. No changes were observed in sleep oscillations. Otoconia-deficient tilted mice which cannot encode linear acceleration due to deficits in the vestibular system, were insensitive to the sleep-promoting effect of rocking at 1 Hz, confirming that the effect is mediated through the vestibular system. The maximal linear acceleration applied was a more important variable than the rocking rate per se. In human studies, lying in a rocking bed (0.25 Hz) during a short nap (Bayer et al., 2011) or over the whole night (Perrault et al., 2019) accelerated sleep onset, entrained NREM sleep oscillations (0.5–5 Hz slow wave activity and sleep spindles) and enhanced sleep-dependent memory consolidation, an effect correlated with the increase in fast sleep spindles (Perrault et al., 2019). Thus, rocking may be an effective way to promote sleep and NREM sleep oscillations which are important for sleep-dependent memory formation.

Another form of vestibular stimulation is the non-invasive form of vagal stimulation. Transcutaneous auricular vagal nerve stimulation consists of injection of thermal current to the external ear canal, which affects the density of endolymph in the inner ear and thereby alters the discharge rate of the vestibular nerve. In turn, vestibular output pathways modulate the activity of brainstem ascending arousal systems (Mercante et al., 2018). Thus, this form of stimulation can alter arousal. Auricular stimulation has been investigated as a potential treatment for insomnia, although there are considerable inter-individual differences in responsiveness (Jiao et al., 2020).

### 7.6. Light stimulation

One of the seminal advances in sleep and circadian research was the discovery of a distinct subset of retinal ganglion cells (intrinsically photosensitive) which express the photopigment melanopsin and signal to the central pacemaker of circadian rhythms, the suprachiasmatic nucleus (Foster et al., 2020). These melanopsin cells are particularly sensitive to the blue range of the light spectrum, which is most prominent in the mornings. This finding led to considerable research which indicated that blue light emitted from streetlights, houselights and particularly from screens held close to the eye could disrupt sleep when exposure was substantial during evening or the night-time (Heo et al., 2017; Tam et al., 2021). Accordingly, red-shifting lights and screens or the use of blue-blocking glasses promotes healthy sleep and circadian rhythms (Wirz-Justice, 2010). Conversely, bright blue light early in the day is beneficial for arousal and subsequent night-time sleep, as well as for mood, especially in those with seasonal affective depression or those living far from the equator in the wintertime (Arendt, 2012; Burns et al., 2021; Wirz-Justice, 2010). Morning blue light also improved daytime sleepiness and quality of life in a small, randomized clinical trial of patients with mild TBI (Raikes et al., 2020).

### 7.7. Gamma frequency (40 Hz) stimulation during wakefulness

Brain-activated states typically exhibit an increase in the power of high-frequency EEG activity, in the beta (15–30 Hz) and gamma (30–80 Hz) bands, mediated by enhanced activity of arousal systems in the basal forebrain (Anaclet et al., 2015; Kim et al., 2015) and thalamus (Steriade et al., 1996). Furthermore, basic science studies have shown that increases in cortical activity during wakefulness lead to an increase in sleep oscillations in the more active area (Krueger and Tononi, 2011). Thus, manipulations which broadly increase brain activity might be expected to increase subsequent sleep. Intriguing recent studies in transgenic mice carrying Alzheimer’s disease related pathological genes suggest that an increase in synchronized 40 Hz activity induced by 40 Hz light pulses and/or auditory stimuli may have beneficial effects for the clearance of proteins linked to pathology in Alzheimer’s disease and improve cognition (Addaikkan et al., 2019; Iacarino et al., 2016; Martorell et al., 2019). Direct 40 Hz stimulation of subcortical arousal systems, such as basal forebrain parvalbumin neurons (Kim et al., 2015; Hwang et al., 2019) might also be effective and is being tested. One possible way that 40 Hz stimulation may improve pathology and cognition is by improving sleep. In a small recent clinical study 14 patients with mild to moderate AD showed improved sleep as assessed by actigraphy measures following 1 h of 40 Hz sensory stimulation/day for 6 months versus 8 sham control subjects (Cimenser et al., 2021). Furthermore, in contrast to the sham stimulation control group which declined in their ability to carry out daily living activities, the stimulation group maintained their ability to function. Thus, 40 Hz stimulation appears a promising avenue to treat sleep disruption in dementia patients. Along the same lines, the rapid antidepressant, ketamine, increases broadband cortical beta and gamma activity (Kocsis et al., 2013) and normalizes sleep and circadian rhythms abnormalities in depressed patients, concurrent with its mood boosting effect (Song and Zhu, 2021). It will be interesting to examine whether pharmacological or stimulation paradigms which enhance cortical gamma activity might also prove beneficial in TBI or other disorders involving sleep disruption.

### 7.8. Challenges and opportunities with non-invasive stimulation approaches

All the stimulation methods described in this section have the potential to alter sleep, arousal and the frequency and amplitude of endogenous brainwaves in the cerebral cortex. In the case of top-down, cortical stimulation, the effects are thought to also reach subcortical arousal networks through cortico-thalamic feedback loops (Krone et al., 2017). In contrast, sensory stimulation paradigms influence thalamocortical oscillations through ascending brainstem and hypothalamic pathways (Bellesi et al., 2014). The challenge that remains to make use of this technology and improve sleep for both healthy individuals and people with sleep disorders is to establish a solid framework for non-invasive brain stimulation. Among others, some key variables that still need to be investigated to determine how to use non-invasive stimulation in the most safe and effective way are stimulation duration, targeted brain regions, optimal time of stimulation, as well as stimulation intensity and frequency (Box 1). TMS and ultrasound techniques, while potentially powerful, are currently not feasible to be applied at home. However, they might still be usefully applied in a hospital setting or optimally in an outpatient clinic setting. Home TES devices exist, as do techniques based on sensory stimulation. However, the optimal stimulation variables still need to be determined and refined. Furthermore, for those techniques which involve modulation of sleep oscillations, ideally the stimuli need to be deployed at the correct phase of ongoing oscillations so further engineering and software advances in real-time sleep oscillation detection are needed for these techniques to be deployed widely and effectively. With the increased deployment of wearable technologies, it would appear these advances will not be too long in coming.

## 8. Invasive approaches (Fig. 2)

Invasive approaches are unlikely to be warranted in the most common sleep disorders. However, they may be considered in sleep disorders caused by neurodegeneration i.e. narcolepsy and REM sleep behavior disorder (Blanco-Centurion et al., 2013; K. Liu et al., 2017; M. Liu et al., 2017; McKenna and Peever, 2017). Invasive approaches may also prove useful in modulating arousal and sleep in other severe neurodegenerative or psychiatric disorders. Invasive approaches are already approved or being tested in these disorders, but sleep is often an afterthought.

### 8.1. Deep brain electrical stimulation (DBS)

Surgical approaches which implant DBS electrodes are already being tested in Parkinson’s disease, other movement disorders and in dementia and could have a role in chronic, severe and treatment refractory insomnia, excessive daytime somnolence and in TBI. Typically, electrodes are implanted in basal ganglia nuclei, in the brainstem pedunculopontine region and in the basal forebrain. Stimulation is generally applied during the day with the goal to improve movement and/or cognition. However, these brain sites also regulate sleep-wake states. Thus, stimulation during daytime may be beneficial in alleviating daytime sleepiness whereas night-time stimulation to improve sleep might also be feasible.

An increasing number of deep brain stimulation studies have targeted basal ganglia nuclei to try to improve sleep abnormalities in Parkinson’s disease such as insomnia, sleep fragmentation and excessive daytime sleepiness (EDS) (Eugster et al., 2016). The majority of these studies targeted the subthalamic nucleus and reported generally positive results on sleep parameters using subjective and in fewer cases also using polysomnography (Eugster et al., 2016; Sharma et al., 2018; Zuzuarregui and Ostrem, 2020). Less prominent effects were observed on EDS. There is evidence that both daytime and night-time stimulation can improve sleep quality. Fewer studies targeted the globus pallidus pars interna, ventral intermediate nucleus of the thalamus or the pedunculopontine nucleus in the brainstem but these studies also appear promising (Eugster et al., 2016; Sharma et al., 2018; Zuzuarregui and Ostrem, 2020). Unlike the other nuclei targeted, stimulation of the pedunculopontine nucleus increased REM duration, consistent with animal studies which implicate this region in REM promotion (Brown et al., 2012; McCarley, 2007). To the best of our knowledge, no study has yet attempted closed-loop DBS to align the stimulation with sleep phases or oscillations, although this appears a promising approach.

The basal forebrain region is an important sleep-wake control region best known for its cortically-projecting cholinergic neurons but also containing substantial numbers of GABAergic and glutamatergic neurons which modulate sleep, wakefulness, cortical electrical activity and cognition (Anaclet et al., 2015; Lin et al., 2015; Xu et al., 2015; Yang et al., 2017). Degeneration of basal forebrain cholinergic neurons is a common feature of dementia in Alzheimer’s disease, Parkinson’s disease, and dementia with Lewy bodies, which likely contributes to functional deficits via loss of cholinergic influences on the cortex as well as loss of effects on neighboring GABAergic and glutamatergic neurons (Yang et al., 2014, 2017; Zant et al., 2016). Consistent with its role as a wake-promoting region, loss of neurons in the nucleus basalis region of the basal forebrain in dementia with Lewy bodies is highly correlated with daytime sleepiness (Kasanuki et al., 2018). Thus, DBS of the nucleus basalis could potentially alleviate daytime sleepiness in dementia patients. A single-subject study reported improved attention, concentration and alertness with 20 Hz DBS of the NBM in a patient with Parkinson-dementia syndrome (Freund et al., 2009). Another DBS study of the nucleus basalis at 20 Hz in Parkinson’s disease subjects led to a small improvement in daytime sleepiness but this effect was not significant in this small study of 6 patients (Gratwicke et al., 2017). There was, however, a reduction in hallucinations, another feature linked to basal forebrain dysfunction (Barrett et al., 2018).

There are several important variables which should be considered for DBS including the target region, the stimulation frequency and the timing and duration of the stimulation. For instance, the basal forebrain is a heterogeneous region, and the percentage of different cell-types varies across the subnuclei. Furthermore, different cell-types discharge at different rates and with different patterns across the sleep-wake cycle. Cholinergic neurons tend to discharge at slower frequencies (<10 Hz), whereas GABAergic and glutamatergic neurons can discharge faster and entrain fast cortical oscillations important for cognition (Kim et al., 2015; Yang et al., 2017). Electrical stimulation at different rates will impact different neuronal cell types differently. Similar considerations apply to other DBS sites. In the future it is possible that optogenetic or chemogenetic techniques may be used to specifically increase or decrease the activity of distinct cell-types within these regions (see Section 8.3.).

### 8.2. Vagus nerve stimulation

Stimulation of the vagus nerve is widely used in a variety of neuropsychiatric disorders such as epilepsy, depression and migraine (Collins et al., 2021). Invasive stimulation involves surgical implantation of a vagus nerve stimulator. A recent study in mice found that vagus nerve stimulation induced widespread cortical and behavioral activation including pupil dilation, locomotion and increased activity of cholinergic and noradrenergic axons in the cortex (Collins et al., 2021) and in the future could be considered for EDS. Thus, invasive vagal nerve stimulation can be considered one way to increase arousal and/or awareness (Thibaut et al., 2019).

### 8.3. Viral vector mediated and transplantation approaches

Pre-clinical researchers now routinely use viral vectors, especially adeno-associated viral (AAV) vectors, to deliver various molecules to defined cell types in the brain to modulate their activity. As with DBS approaches, viral vector-mediated gene transfer and transplantation approaches are already being tested in neurodegenerative disorders (Hudry and Vandenberghe, 2019) and could potentially be adapted to treat sleep in these disorders, including the sleep disorders, narcolepsy (K. Liu et al., 2017; M. Liu et al., 2017) and REM sleep behavior disorder (McKenna and Peever, 2017). Currently, viral vectors are directly injected into the brain but progress is being made in developing ways to infect specific brain areas following peripheral injection of viral vectors (Bedbrook et al., 2018; Chan et al., 2017;). Focused ultrasound can also be used to transiently enhance blood-brain permeability and facitate AAV transduction of neurons and astrocytes in the target area (Thévenot et al., 2012) for neuromodulation using chemogenetic (Szablowski et al., 2018) or optogenetic techniques (Wang et al., 2017). Modification of the protein coat surrounding viral DNA (Chan et al., 2017) or the use of a specific promoter or enhancer can allow selective targeting of particular types of neurons such as orexin/hypocretin (Adamantidis et al., 2007) or parvalbumin neurons (Vormstein-Schneider et al., 2020), including in primates (Stauffer et al., 2016; Vormstein-Schneider et al., 2020) and human brain tissue (Vormstein-Schneider et al., 2020).

One widely used technique used in preclinical research is optogenetics, a method to increase or decrease the activity of target neurons through expression of light-sensitive ion channels or pumps (Deisseroth, 2015). The first application of this technique to the sleep field showed that stimulation of orexin/hypocretin neurons in mice could promote wakefulness (Adamantidis et al., 2007). Subsequent work showed the potential to increase sleep or cortical oscillations typical of wakefulness or sleep (e.g. Kim et al., 2015; Thankachan et al., 2019). Recent work has shown that cell-type specific optogenetics in primates is feasible (Stauffer et al., 2016). Furthermore, the first application of optogenetics to a human disorder was recently reported in a case of retinitis pigmentosa (Sahel et al., 2021). Optogenetic stimulation of the retina partially restored sight in this patient. Disturbances of circadian rhythms and sleep are common in patients with retinal dysfunction (Schmoll et al., 2011). Thus, this type of treatment might also prove beneficial in correcting these symptoms via activation of melanopsin-containing retinal ganglion cells (Venner et al., 2019). Optogenetic therapy has not yet been successfully applied to the brain in humans but will likely be useful in disorders where other invasive techniques are already being attempted. One potential issue with optogenetic stimulation is the need to implant optical fibers into the brain for long-term stimulation of the target neurons. A potential solution to this issue is the development of opsins with high-light sensitivity which can be activated via transcranial optical stimulation (Gong et al., 2020). Another solution could involve another cutting-edge technique widely used in preclinical studies, chemogenetics, which modulates the activity of neurons through expression of modified G-protein coupled receptors which are activated by otherwise inert compounds. Recent studies have shown the feasibility of using this technique in primates (Rosebloom et al., 2021). When combined with delivery of viral vectors to the brain via blood-brain barrier opening, chemogenetics has the potential to be completely non-invasive (Szablowski et al., 2018).

The sleep disorder narcolepsy typically involves degeneration of orexin/hypocretin neurons in the perifornical hypothalamus. Pioneering pre-clinical work by Shiromani and colleagues suggests that transplantation or gene therapy approaches using viral vectors may represent a potential avenue to treatment (Blanco-Centurion et al., 2013; Liu et al., (2008, 2017). Similar approaches may be valuable to treat the destruction of brainstem muscle atonia neurons which results in RBD (Gagnon et al., 2006; McKenna and Peever, 2017).

### 8.4. Gene editing techniques

Gene manipulation techniques are now widely used in basic science research investigating sleep-wake circuitry. For instance, techniques such as gene knockouts (Chemelli et al., 1999), RNA interference (Chen et al., 2010) and optogenetics (Adamantidis et al., 2007) proved pivotal in understanding the function of the orexin/hypocretin system and its postsynaptic targets. The Nobel Prize winning CRISPR technique has recently been used to investigate sleep-wake circuitry (Yamaguchi et al., 2018; Yamaguchi and De Lecea, 2019) and thalamocortical oscillations (Uygun et al., 2022) for the first time. CRISPR forms the basis of a technology that can be used to precisely edit genes within organisms. This technique has also been used clinically already. However, most sleep-wake disorders have a complex genetic involvement. Nonetheless, gene editing techniques might prove useful for rare monogenic disorders or to alleviate sleep spindle abnormalities in severe neuropsychiatric conditions such as schizophrenia (Ghoshal et al., 2020).

## 9. Translational approaches to influence different aspects of sleep

Different sleep features are abnormal in various neuropsychiatric disorders (Krystal, 2019). Thus, approaches to selectively modify individual sleep features are desirable. In this section we integrate the previous sections to discuss the most promising approaches to influence specific aspects of sleep and wakefulness.

### 9.1. Stimulation during wakefulness to enhance subsequent sleep

Leading a physically and mentally active lifestyle has many benefits for health, including improved sleep. Extended wakefulness leads to an increased homeostatic drive to sleep. Similarly, a large body of work suggests that an increased intensity of brain activity during wakefulness may lead to local increases in the propensity or need for sleep (Krueger and Tononi, 2011). Accordingly, methods which increase neural activity during wakefulness, such as TMS, may increase slow-wave activity in subsequent sleep (Huber et al., 2007). In a recent study, 40 Hz visual and auditory stimulation benefitted sleep in a small sample of Alzheimer’s disease patients (Cimenser et al., 2021). Similar stimulation improved pathology in Alzheimer’s disease mouse models (Addaikkan et al., 2019; Iacarino et al., 2016; Martorell et al., 2019). High-frequency (> 130 Hz) deep brain stimulation of basal ganglia nuclei during wakefulness also benefits subsequent sleep in Parkinson’s disease patients (Eugster et al., 2016; Sharma et al., 2018; Zuzuarregui and Ostrem, 2020). Pharmacologically increasing brain activity using ketamine in patients with monopolar depression had beneficial effects on sleep (Song and Zhu, 2021). Thus, inducing high-frequency EEG activity during wakefulness via pharmacological, non-invasive or invasive stimulation methods may be a useful way to improve sleep and hinder the build-up of toxic proteins implicated in neurodegeneration.

### 9.2. Initiation of sleep

Approaches to initiate sleep include CBT-I, allosteric GABA_A_ receptor agonists, orexin receptor antagonists, melatonin agonists, electrical, magnetic or vestibular stimulation. Regarding pharmacological treatments, GABA_A_ receptor agents are generally more effective in reducing sleep onset latency when compared to orexin receptor antagonists (Herring et al., 2019). Non-invasive brain stimulation has shown some promise in accelerating sleep onset in healthy subjects, as well as in patients with sleep disorders. As the brain transitions to the first stage of NREM sleep, different brain regions go through distinct and temporally specific changes in neural activity (Marzano et al., 2013; Prerau et al., 2017). First, EEG signals across all cortical areas show a synchronized increase in low-frequency activity in the 0.50–7 Hz range, and a parallel decrease in activity in the beta (18–25 Hz) frequency range. Second, the slow waves (0.75–0.4 Hz) that are key indicators of sleep show an antero-posterior gradient in their temporal propagation across the brain: slow wave activity (SWA) first appears in the prefrontal cortex before propagating to posterior regions. Third, theta activity (4 – 8 Hz) shows a temporo-occipital diffusion in a similar timeframe to the antero-posterior diffusion of SWA (Marzano et al., 2013). These findings don’t paint a complete picture of the entire sleep onset process, but they provide valuable indicators of which brain regions could be targeted by non-invasive stimulation techniques to “jumpstart” this transition to sleep. Researchers who have investigated the sleep-inducing potential of non-invasive brain stimulation techniques have therefore adopted a top-down approach that aims to expedite the transitions described above by enhancing SWA and theta activity in frontal regions, but also more globally across the cortex.

Currently, targeting frontal brain regions with an anodal current alternating at 5 Hz appears to be the most effective choice to induce sleepiness with tES (D’Atri et al., 2016).

Studies which focus on using non-invasive brain stimulation to facilitate the onset of sleep in individuals with sleep disorders are rare and have low sample sizes. In a poster abstract Jung and Jun (2019) describe how a month-long tDCS treatment decreased sleep onset latency in people with insomnia. 7 participants were split into 3 groups such that 3 received anodal stimulation, 2 received cathodal stimulation, and 2 received sham stimulation. They found improvements in sleep onset latency in 2 out of the 3 participants in the anodal group, 1 of the 2 in the cathodal group, and 0 out of 2 in the sham group (Jung and Jun, 2019). However, they did not mention whether these findings were found through objective measurements or subjective reporting. Although the sample size for each condition group in this study was very small, these results can be used as preliminary evidence that tDCS treatments could help insomnia patients fall asleep faster, after further research. Similarly, Kunze et al. (2007) in two case reports briefly describe how using rTMS reduced sleep onset latency for 2 individuals with insomnia. rTMS was delivered across 15 sessions on the dorsolateral prefrontal cortex, with one participant receiving 1 Hz stimulation on the right DLPFC, and the other receiving 10 Hz rTMS on the left DLPFC (Kunze et al., 2007). Once again, little information is available and the sample size of this study was very small, but these preliminary findings suggest that rTMS might help people with insomnia fall asleep faster.

### 9.3. Sleep continuity

Sleep fragmentation is a common feature of many sleep disorders and neuropsychiatric conditions. Sleep fragmentation reduces deep NREM and REM sleep, impairing the restorative effects of these sleep stages and increases daytime sleepiness (McKenna et al., 2007). In animal studies, sleep fragmentation mimicking that seen in sleep apnea reduces neuronal excitability and impairs hippocampal synaptic plasticity and declarative memory formation (Tartar et al., 2006, 2010). Human studies also report that sleep fragmentation impairs off-line consolidation of motor memories (Djonlagic et al., 2012). In newly diagnosed sleep apnea patients, deficits in sleep-dependent memory formation were correlated with reductions in the duration of NREM stage 3 sleep and these deficits could be reversed by CPAP treatment (Djonlagic et al., 2021b). In older adults, sleep duration and continuity were major determinants of cognitive deficits (Djonlagic et al., 2021a). Deep stages of NREM sleep with high slow wave activity are also linked to glymphatic clearance of toxic proteins in animals (Xie et al., 2013) and in humans (Fultz et al., 2019). Thus, therapeutic approaches which improve sleep continuity and maintenance may prove particularly beneficial. Amongst pharmacological agents, orexin receptor antagonists appear to be more effective in promoting sleep maintenance than GABA_A_ receptor agonists (Herring et al., 2019). Other pharmacological agents such as melatonin receptor agonists or tricyclic antidepressants may also be effective (Krystal, 2019).

Internally or externally generated sensory stimuli are a major contributor to disrupted sleep continuity. Pain is a major contributor to poor sleep and on the other hand, sleep loss heightens pain. Loud auditory stimuli such as snoring, sirens etc., are common sleep disruptors in otherwise healthy people. Unpleasant sensations from the extremities disrupt sleep in patients with restless legs syndrome. In obstructive sleep apnea, increases in blood carbon dioxide levels due to closure of the upper airway during sleep lead to a reflex which reopens the airway but also causes an activation of the cortex which disrupts sleep continuity and prevents deep sleep. Recent basic science studies have begun to reveal the pathways responsible for arousals from sleep due to sensory stimuli. The parabrachial nucleus of the brainstem (Kaur et al., 2017; Kaur and Saper, 2019) and serotonergic raphe neurons (Kaur et al., 2020) are particularly important for arousals due to increases in blood carbon dioxide levels (hypercarbia) whereas basal forebrain parvalbumin neurons mediate arousals in response to both hypercarbia and auditory stimuli (McKenna et al., 2020). Knowledge of these pathways and molecular profiling of the neurons involved may be useful in developing treatments which depress cortical arousals while maintaining important brainstem reflexes.

Non-invasive brain stimulation approaches could also be useful to help people increase the continuity of their sleep, preventing undesired nighttime awakenings and increasing overall sleep time (see Section 7. 1. And 7.2.). Changing cortical activity to enhance slower brain wave activity either right before or during sleep can improve sleep continuity by preventing nighttime awakenings (Annarumma et al., 2018). For people with insomnia specifically, these findings appear logical since decreasing cortical activity could reduce symptoms of hyperarousal, which can in turn prevent sleep fragmentation and lead to more continuous sleep.

### 9.4. Enhancing sleep duration

The propensity to sleep is determined by both circadian and homeostatic factors. Thus, going to bed at the appropriate time of the evening is an important determining factor of how long sleep duration will be. Use of melatonin receptor agonists may be useful in that regard. In general, though, pharmacological agents such as allosteric GABA_A_ receptor agonists and orexinergic antagonists elicit only relatively small increases in overall sleep time vs placebo. Similarly, non-invasive or invasive approaches attempted to date have relatively modest effects on altered sleep duration, with the exception of one study of patients with chronic primary insomnia (Jiang et al., 2013) where the patients were only averaging 5.5 hrs sleep a night under baseline conditions. Generally, it appears that there is a barrier to extending sleep beyond the 7–9 h in humans which is considered a healthy sleep duration for most adults (Watson et al., 2015). These findings raise the questions of what mechanisms limit maximal sleep duration in adults; and whether these mechanisms can be overcome to maximally activate the restorative effects of sleep (Box 1).

### 9.5. Enhancement of NREM oscillations: sleep spindles, delta waves and slow oscillations

Beyond accelerating sleep onset and preventing nighttime wakefulness, improving the sleeping experience also involves optimizing sleep architecture. Different neuropsychiatric disorders have distinct profiles in terms of sleep architecture (Ferrarelli, 2021; Krystal, 2019; Manoach and Stickgold, 2019). Furthermore, different sleep features have been associated with distinct functions. Thus, it may prove beneficial to be able to target specific sleep oscillations. GABAergic hypnotics such as zolpidem and eszopiclone tend to increase sleep spindles, at the expense of slow wave activity. Thus, several studies have attempted to increase sleep spindles and improve sleep-dependent memory consolidation in healthy participants (Mednick et al., 2013; Zhang et al., 2020) and in patients with schizophrenia (Wamsley et al., 2013) through application of the GABAergic hypnotics. Positive effects on memory consolidation have been reported in healthy participants, but so far the effects on memory in schizophrenia patients have been disappointing, likely because sleep spindles need to be coupled with the correct phase of cortical slow oscillations and allosteric modulators of GABA_A_ receptors tend to reduce slow oscillations (Manoach and Stickgold, 2019; Mylonas et al., 2020).

Non-invasive brain stimulation techniques may be more promising than pharmacological methods for boosting NREM sleep oscillations due to their ability to precisely time stimulation and induce oscillations at defined frequencies (Frohlich and Lustenberger, 2020). TES, TMS and acoustic stimulation have all been used to enhance NREM sleep oscillations (Bellesi et al., 2014; Feher et al., 2021; Helfrich et al., 2014; Herrero Babiloni et al., 2021) Jiang et al., 2013; Krone et al., 2017; Malkani and Zee, 2020; Massimini et al., 2007; Ngo et al., 2017; Saebipour et al., 2017). Increasingly, closed-loop stimulation approaches are coupling stimulation to particular phases of sleep or sleep oscillations, which is likely to improve their effectiveness given current models of the architecture of NREM sleep which emphasize the importance of coupling of NREM oscillations throughout the forebrain (Crunelli and Hughes, 2010; Rasch and Born, 2013).

### 9.6. Enhancement of REM sleep/REM sleep oscillations

Although the function of REM sleep is unknown, one possible role is manipulation of emotional memories (Walker and Van der Helm, 2009). Abnormal REM sleep has been implicated in the pathogenesis of insomnia (Riemann et al., 2012; Van Someren, 2021), affective, anxiety and autistic disorders (Baglioni et al., 2016; Benca et al., 1992) and especially in PTSD (Ross et al., 1989; Germain, 2013; Kaplan et al., 2022). Accordingly, there is interest in treatments which might selectively manipulate REM sleep or REM sleep oscillations. A major function of the orexin/hypocretin system is suppression of REM sleep and loss of this system leads to aspects of REM sleep intruding into wakefulness. Thus, it is perhaps not surprising that orexin receptor antagonists increase the amount of REM sleep in animal models and in humans (Herring et al., 2019). Fortunately, they do not produce the pathological REM-related narcolepsy symptoms such as cataplexy in humans at the approved doses [Note: cataplexy has been observed in mice with very high doses of orexin receptor antagonists and conditions where orexin levels are reduced] (Kaushik et al., 2021). Thus, application of dual orexin receptor antagonists, particularly at higher doses, is one possible way to enhance REM sleep and treat disorders such as PTSD (Kaplan et al., 2022). Increasing MCH neuronal or receptor activity would also be expected to enhance REM sleep (see Section 6.6.) whereas serotonin and noradrenaline re-uptake inhibitors strongly suppress REM sleep and noradrenaline receptor antagonists such as prasozin might be beneficial in suppressing disturbing dreams.

Recent animal findings suggest that another potential way to boost entry into REM sleep could be via manipulation of the thalamic reticular nucleus and sleep spindle activity, since optogenetic enhancement of sleep spindles increased entry into REM sleep (Bandarabadi et al., 2020). Enhancement of REM sleep has generally not been a specific focus of non-invasive brain stimulation approaches. However, Jiang and colleagues (2013) found that repetitive TMS of dorsolateral prefrontal cortex at 1 Hz for 30 min/day for 2 weeks in patients with chronic primary insomnia increased REM sleep duration more than pharmacological or psychotherapy treatments. In contrast, Saebipour et al. (2015) found no effect in REM sleep duration when using oscillating 0.75 Hz tDCS during stage 2 sleep in insomnia patients. Interestingly, gamma frequency (25 or 40 Hz) tACS stimulation of frontal cortex during REM sleep led to self-awareness during dreams in healthy volunteers i.e. lucid dreaming (Voss et al., 2014). In another fascinating study, a combination of machine-learning and functional magnetic resonance imaging was used to decode visual imagery during the sleep-onset period (Horikawa et al., 2013). Potentially, these approaches could be applied to dreams in both NREM and in REM periods and suggests the tantalizing possibility of using non-invasive stimulation approaches to directly modify disturbing dreams in disorders such as PTSD (Krone et al., 2017).

### 9.7. Enhancement or suppression of sleep-dependent memories

Memory consolidation is a commonly discussed function of sleep (Rasch and Born, 2013; Stickgold, 2005). Despite one hundred years of research, this potential function of sleep remains controversial, and arguably it may not be the most clinically relevant aspect of sleep, except in dementia patients. Nonetheless, there is enormous interest in trying to boost sleep-dependent memory consolidation through pharmacological or brain stimulation techniques. As discussed in Section 6, GABAergic agonists may promote memory consolidation by enhancing sleep spindle density whereas a high-profile basic science study suggested a role for MCH neurons in forgetting hippocampus-dependent learning and modulating fear conditioning. Thus, pharmacological approaches to enhance or suppress sleep-dependent memory formation may be possible. With regards to brain stimulation approaches, enhancement or suppression of sleep-dependent memories can be achieved through acoustic, electrical or magnetic enhancement of sleep oscillations and some prominent examples are provided next.

Marshall et al. (2006) were the first to modulate sleep structure with the goal of improving declarative memory in healthy subjects. They applied osc-tDCS to frontal brain regions for 5 min at a frequency of 0.75 Hz during stage 2 of NREM sleep and found an increase in SWA in the frontal cortex as well as improved declarative memory in a word recall task compared to sham. They found no such effects when stimulating at 5 Hz with all other experimental variables being the same (Marshall et al., 2006). More recently, Ladenbauer and colleagues (2017) used a similar slow osc-tDCS paradigm in patients with mild cognitive impairment. Stimulation was applied during a daytime nap in a sleep-state-dependent manner to modulate sleep oscillations and sleep-related memory consolidation in nine male and seven female human patients. Stimulation significantly increased overall slow oscillation and spindle power, amplified spindle power during SO up-phases, and led to stronger synchronization between SO and spindle power fluctuations in EEG recordings. Moreover, visual declarative memory was improved by so-tDCS compared with sham stimulation and was associated with stronger synchronization.

Similarly, Lustenberger et al. (2016) examined the possibility of increasing sleep spindles using tACS to subsequently improve motor memory. Their tACS montage delivered short bursts of 12 Hz current which had spindle-like waveforms on participants’ frontal lobe once real-time analyses determined that subjects were in a NREM sleep phase where spindles were already occurring naturally. They found no significant changes in sleep architecture due to the stimulation, but an increase in spindle activity between 11 and 16 Hz in stage 2 of NREM sleep across the cortex (Lustenberger et al., 2016). To investigate the correlation between the effect on spindle activity and memory, participants completed declarative and procedural memory tasks before going to sleep and after waking up. Subjects who saw an increase in fast spindle activity (in the 15–16 Hz range) had shorter response times in the motor memory task (Lustenberger et al., 2016), suggesting that the density and duration of fast spindles during stage 2 of NREM sleep is an important determining variable for motor memory consolidation. tACS could therefore be a useful tool to improve memory consolidation by inducing sleep spindles.

Finally, Cellini et al. (2019) investigated the possibility of improving declarative memory of facts using a tACS montage which delivered a 0.75 Hz stimulation to four frontal regions of the brain. Short stimulation bursts of 4 s were triggered in real time when slow oscillations were detected by EEG electrodes while the participants napped. tACS improved performance in the declarative memory task (Cellini et al., 2019), revealing that it can reliably improve declarative memory even when used during daytime naps. These outcomes on memory consolidation show that timing of stimulation is a crucial variable for memory consolidation, especially since Kirov et al. (2009) found no improvement in memory consolidation when stimulating participants with tACS while they engaged in a memory encoding task before going to sleep.

### 9.8. Enhancement of sleep-dependent clearance of toxic proteins

One of the most exciting advances in the sleep field in recent years is the discovery that the levels of toxic proteins implicated in neurodegenerative diseases vary according to sleep-wake state (Kang et al., 2009) and their clearance is regulated by the so-called ‘glymphatic system’ (Rasmussen et al., 2022). This system appears to be most active during deep NREM sleep with high slow-wave activity in animals (Xie et al., 2013) and in humans (Fultz et al., 2019). Thus, manipulations which boost deep NREM sleep may be beneficial for treating people with brain injuries or neurodegenerative conditions. On the other hand, it may also be conceivable to boost the activity of this system independently of effects on deep NREM sleep. Intriguingly, a rodent study showed that low-intensity ultrasound targeting the hippocampus increased glymphatic function, leading to enhanced drainage of beta-amyloid from the brain to CSF in an amyloid overexpressing mouse model of Alzheimer’s disease (Lee et al., 2020). However, stimulation was performed under anesthesia not during sleep in this study. Thus, specific glymphatic boosting strategies during sleep are not yet tested.

### 9.9. Regulation of metabolism

A large body of work links short or disrupted sleep to gain in weight and to cardiovascular disorders (Javaheri and Redline, 2017). Thus, manipulation of sleep and arousal can be considered as potential avenues to promote healthy weight, metabolic and cardiovascular function. The various neurons of the lateral hypothalamus are promising targets since several different neuronal subtypes in this region respond to both metabolic and sleep regulatory signals and influence both functions (Arrigoni et al., 2019; Oesch and Adamantidis, 2021). Orexins were originally named by one group due to their hypothesized role in regulating appetite. Now it is recognized that sleep-wake control is their primary function, thus their other name, hypocretins, may be more appropriate, but they still likely play a role in autonomic activity and body weight control (Willie et al., 2001). Orexins/hypocretins and other lateral hypothalamic peptides affect the activity of ventral tegmental area dopamine and non-dopaminergic neurons, which themselves have a dual role in regulating arousal and food intake (Korotkova et al., 2003, 2006). More recently, melanin-concentrating hormone and GABAergic lateral hypothalamic neurons have been a focus of attention since they control both feeding and sleep (Arrigoni et al., 2019; Oesch and Adamantidis, 2021). Thus, pharmacological manipulation of these lateral hypothalamic pathways may be a promising approach for metabolic disorders, through sleep manipulation. Brain stimulation approaches to enhance sleep quality and duration are also likely to be effective.

## 10. Conclusions

A century of basic science research in the sleep-wake and circadian fields has uncovered the brain regions, neurotransmitters, proteins, and genes which control sleep, wakefulness and the cortical electrical oscillations which accompany these states in mammals (Brown et al., 2012). These basic science advances have contributed to translational approaches in several different ways. Theoretical models of how sleep amount and timing are controlled underpin behavioral approaches to the management of sleep disorders. The discovery of new neurotransmitter systems/receptors has proven particularly fruitful in developing new pharmacological approaches targeting the brain histamine and orexin/hypocretin systems and have refined our understanding of the GABA_A_ receptor subunits which are responsible for the hypnotic actions of allosteric GABA_A_ receptor antagonists. Basic science work which revealed the brainstem, hypothalamic and basal forebrain circuitry underlying arousals from sleep in response to hypercarbia or auditory stimuli hold the promise to improve sleep continuity by dampening the responsivity of those pathways (Kaur et al., 2017; Kaur and Saper, 2019; McKenna et al., 2020). Identification of the genes which regulate sleep and arousal opens up the possibility of precision pharmacogenetics (Krystal and Prather, 2019), diagnosis of circadian rhythms disorders and natural extremes in sleep-wake amount (Ashbrook et al., 2020; Dashti et al., 2019). For instance, polymorphisms in adenosine related genes affect sensitivity to the arousing properties of caffeine, sleep depth and the detrimental effects of sleep deprivation on cognition (Bachmann et al., 2012; Bodenmann et al., 2012). Basic science work on the mechanisms and functions of sleep oscillations have initiated attempts to manipulate them using brain stimulation approaches. Cutting-edge neuroscience techniques suggest the possible to manipulate sleep and arousal circuitries deep in the brain either invasively or non-invasively. Nonetheless, developing this knowledge into translational success stories requires the combined efforts of neuroscientists, clinicians, engineers, mathematical modelers, chemists as well as continued financial support from public and private sources to achieve the ultimate goal of developing new treatments for the myriad disorders of sleep and arousal which affect humanity.

## Figures and Tables

**Fig. 2.**
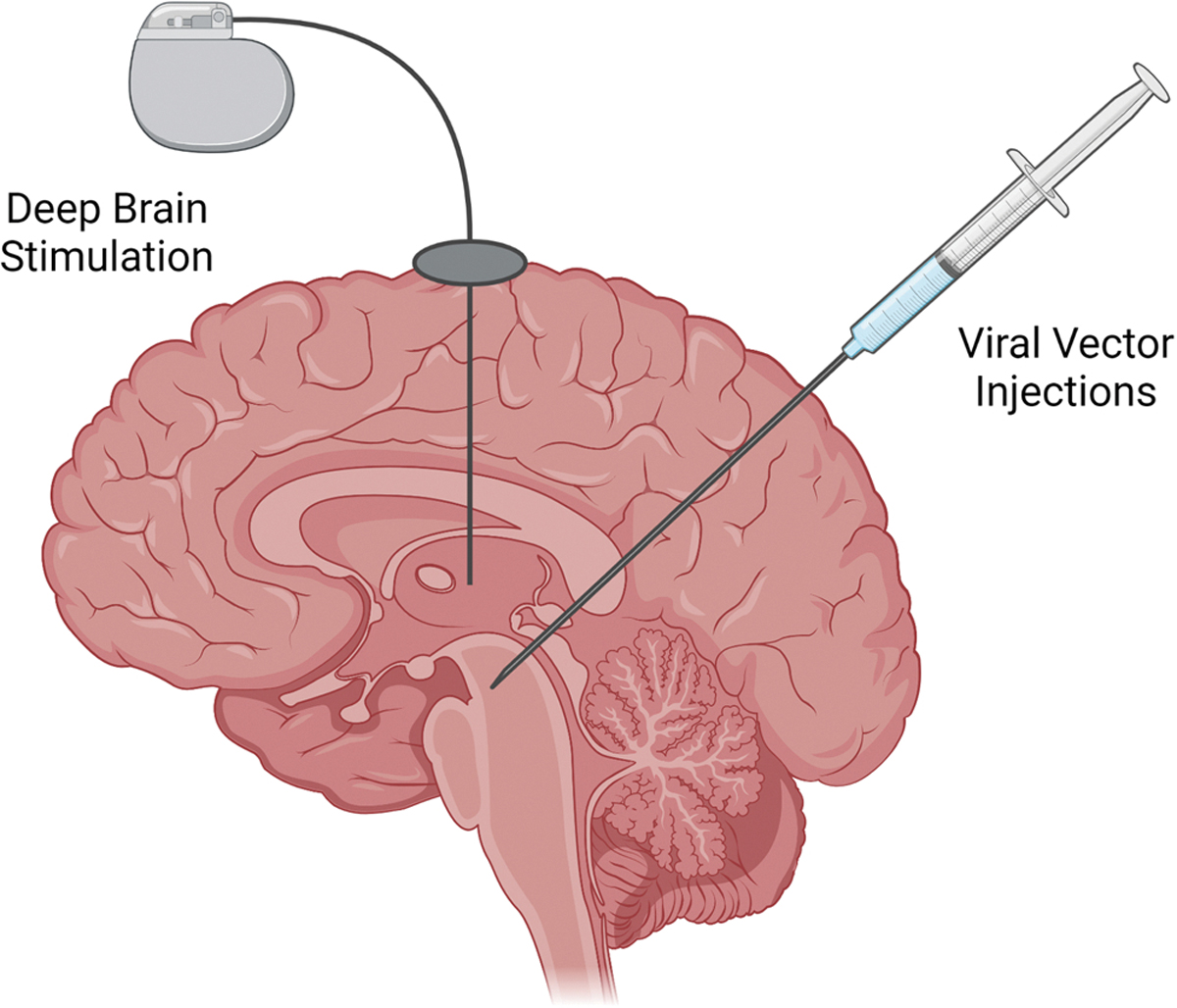
Invasive approaches to modulate sleep and arousal may be warranted in severe neurological and psychiatric disorders. Deep brain stimulation is already in use in basal ganglia degenerative disorders and shows promise in improving sleep. Basic science studies use viral vectors injected into specific brain regions to modulate the activity of neurons by optogenetic, chemogenetic or gene editing techniques. These techniques are being tested in primates for future use in humans. Transplantation techniques for neurodegenerative conditions such as narcolepsy and RBD are also feasible. Figure created with BioRender.com.

## Box 1 Questions for future translational approaches.

1. Can we predict which individuals are likely to be helped most by behavioral, pharmacological, non-invasive or invasive approaches based on baseline sleep/arousal measures or genetics?
2. Which treatment approach (behavioral, pharmacological, non-invasive stimulation, invasive stimulation) is most likely to result in sleep improvements that last the longest after the treatment is finished?
3. Which brain areas or cell-types are most effective and ‘targetable’ for modulating sleep and arousal?
4. Can we directly manipulate the activity of sleep-wake control neurons rather than altering their postsynaptic receptors? Can single-cell RNA analysis or other approaches identify genes/proteins which enable selective targeting or is there too much redundancy in the system?
5. Can astrocytes be targeted to modulate sleep in humans?
6. Will top-down modulation of cortical neurons be reliable and effective in modulating sleep, or do we need to target sleep-wake circuits deeper in the brain?
7. What mechanisms limit maximal sleep duration or individual sleep features such as slow-wave activity and can these maximal limits be overcome to further enhance the restorative properties of sleep?
8. What is the physiological role of different GABAergic sleep promoting neurons in the preoptic hypothalamus, lateral hypothalamus, and brainstem? Can we target them individually?

## Abbreviations:

AAV: Adeno-associated viral vector
ATP: Adenosine Triphosphate
CBT-I: Cognitive Behavioral Therapy for Insomnia
CPAP: Continuous positive airway pressure
CRISPR: Clustered regularly interspaced short palindromic repeats
DBS: Deep brain electrical stimulation
DLPFC: Dorsolateral Prefrontal Cortex
DORA: Dual orexin receptor antagonist
EDS: Excessive Daytime Sleepiness
EEG: Electroencephalogram/Electroencephalography
EMG: Electromyogram
FDA: (United States) Food and Drug Administration
GABA: Gamma-Amino-Butyric Acid – major inhibitory neurotransmitter
MCH: Melanin concentrating hormone
NREM: Non-rapid-eye-movement (Sleep)
Osc-tDC: Oscillating transcranial direct current stimulation
PTSD: Post-Traumatic Stress disorder
RBD: REM sleep behavior disorder
REM: Rapid-eye-movement (Sleep)
rTMS: repetitive Transcranial Magnetic Stimulation
tACS: transcranial Alternating Current Stimulation
SOL: Sleep onset latency
SWA: Slow (0.5–4 Hz) wave activity during NREM sleep
TBI: Traumatic brain injury
TC: Thalamocortical (Neurons)
tDCS: transcranial Direct Current Stimulation
TMS: Transcranial Magnetic Stimulation
TRN: Thalamic reticular nucleus
TRP: Transient Receptor Potential family of ion channels
TST: Total sleep time
VA: Veterans Administration
WASO: Wakefulness after sleep onset
